# Categorizing prediction modes within low-pLDDT regions of *AlphaFold*2 structures: near-predictive, pseudostructure and barbed wire

**DOI:** 10.1107/S2059798325007843

**Published:** 2025-09-12

**Authors:** Christopher J. Williams, Vincent B. Chen, David C. Richardson, Jane S. Richardson

**Affiliations:** a Department of Biochemistry Duke University School of Medicine 132 Nanaline Duke Building 3711 DUMC Durham NC27710 USA; Lund University, Sweden

**Keywords:** *AlphaFold*2, structure prediction, conditional folding, structure validation, intrinsically disordered regions, low pLDDT, barbed wire, near-predictive, signal peptides

## Abstract

*AlphaFold*2 protein structure predictions are widely available for structural biology uses. These predictions, especially for eukaryotic proteins, frequently contain extensive regions predicted below the pLDDT = 70 level, the rule-of-thumb cutoff for high confidence. This work identifies major modes of behavior within low-pLDDT regions through a survey of human proteome predictions provided by the AlphaFold Protein Structure Database. The *near-predictive* mode resembles folded protein and can be a nearly accurate prediction. *Barbed wire* is extremely unprotein-like, being recognized by wide looping coils, an absence of packing contacts and numerous signature validation outliers, and it represents a region where the conformation has no predictive value. *Pseudo­structure* presents an intermediate behavior with a misleading appearance of isolated and badly formed secondary-structure-like elements. These prediction modes are compared with annotations of disorder from MobiDB, showing general correlation between *barbed wire*/*pseudostructure* and many measures of disorder, an association between *pseudostructure* and signal peptides, and an association between *near-predictive* and regions of conditional folding. To enable users to identify these regions within a prediction, a new *Phenix* tool is developed encompassing the results of this work, including prediction annotation, visual markup and residue selection based on these prediction modes. This tool will help users develop expertise in interpreting difficult *AlphaFold* predictions and identify the *near-predictive* regions that can aid in molecular replacement when a prediction does not contain enough high-pLDDT regions.

## Abstract 2

Behavioral modes of *AlphaFold*2 predictions at low pLDDT are identified and described, ranging from unprotein-like ‘barbed wire’ to near-predictive folds, and a tool to automate this identification is provided. Associations between prediction modes and features of interest, such as regions of conditional folding and signal peptides, are discussed.

## 1. Introduction

*AlphaFold* structure prediction (Jumper *et al.*, 2021 ▸) has revolutionized experimental design in structural biology (Thornton*et al.*, 2021 ▸) and greatly eased the early stages of experimental structure solution (Terwilliger *et al.*, 2023 ▸, 2024 ▸). High-confidence *AlphaFold* predictions make excellent molecular-replacement targets for X-ray crystallography and starting models for cryoEM. *AlphaFold*’s own pLDDT (predicted Local Distance Difference Test) provides a guide to whether a region is usable for structural biology, with pLDDT of ≥70 serving as a rule-of-thumb cutoff for high-confidence regions and pLDDT ≥ 90 for very high confidence. However, especially in predictions of eukaryotic proteins, many residues fall outside this high-confidence regime.

Besides pLDDT, packing relationships have been recognized as a key feature for identifying regions of interest in *AlphaFold* predictions. *Critical Assessment of protein Intrinsic Disorder* (*CAID*) makes use of the AlphaFold-Bind measure as one of its baselines for identifying intrinsically disordered regions (IDRs) with conditional folding (Conte *et al.*, 2023 ▸), and AlphaFold-Bind depends in part on accessible surface area (Piovesan *et al.*, 2022 ▸). The *AlphaCutter* tool has been developed as an alternative method for preparing *AlphaFold* predictions for downstream structural biology uses, and it uses contact packing to identify and preserve folded regions with potential predictive value even at low pLDDT (Tam & Iwasaki, 2023 ▸).

We previously reported on the presence of such well folded, protein-like regions within low-pLDDT (pLDDT < 70) predictions, calling them ‘near-folded’ in reference to their presumed closeness to the target structure (Richardson *et al.*, 2023 ▸). Other work has supported the predictive value of selected low-pLDDT regions, with residues having pLDDT as low as 40 being useful in constructing molecular-replacement targets (Wang *et al.*, 2025 ▸).

Selective identification of predictive or near-predictive residues is important because low-pLDDT regions are dominated by nonpredictive residues, *i.e.* residues where the atomic coordinates have no relation to the target structure. The most obvious nonpredictive regions are *barbed wire*, which appears highly disordered and hugely enriched in validation outliers. These regions must be removed for many structural biology tasks, including when preparing molecular-replacement targets.

The relationship of *AlphaFold* predictions to disorder has been an area of considerable interest (Conte *et al.*, 2023 ▸; Necci *et al.*, 2021 ▸; Wilson *et al.*, 2022 ▸). Unsurprisingly, low-pLDDT regions correspond strongly with intrinsically disordered regions (Tunyasuvunakool *et al.*, 2021 ▸). However, high-pLDDT residues can also correspond with intrinsic disorder, especially regions of conditional folding (Alderson *et al.*, 2023 ▸). pLDDT alone is not sufficient to identify modes of disorder within *AlphaFold*2 predictions.

This work formally categorizes different behaviors in *AlphaFold*2 predictions, especially the low-pLDDT regions, adding additional modes to our previous classification to make better sense of the ambiguous mid-confidence range pLDDT ∼40–70. We are primarily concerned with identifying modes where the Cartesian coordinates of residues have predictive value. We also explore the relationships between our modes and protein disorder through comparison to disorder annotations from the MobiDB database (Piovesan *et al.*, 2025 ▸) to determine how these modes serve as predictions of sequence character even when they do not predict usable residue coordinates. We present a tool to automatically split an *AlphaFold* prediction into our modes. This tool expands on *AlphaCutter*’s approach by adding *MolProbity* validation metrics (Williams *et al.*, 2018 ▸) in addition to our own version of packing analysis (Davis *et al.*, 2007 ▸). Our tool is written in Python and is included in the *Phenix* software package (Liebschner *et al.*, 2019 ▸) as *phenix.barbed_wire_analysis* and in the *Computational Crystallography Toolbox* (*cctbx*) as *molprobity.barbed_wire_analysis*.

## 2. Methods

### 2.1. Data set

We downloaded the complete human proteome prediction from the AlphaFold Protein Structure Database (Varadi *et al.*, 2024 ▸; initially version 2, and version 4 when it became available; the differences in these versions did not appear to impact our analysis). We chose the human proteome because human proteins contain complex binding, regulation and generally extensive intrinsically disordered regions, making them challenging cases for *AlphaFold* and good test cases for examining low-pLDDT predictions, especially *barbed wire*. The human proteome is also relatively well annotated in MobiDB (see below).

We also downloaded complete proteome predictions for *Escherichia coli*, *Staphylococcus aureas* and *Saccharomyces cerevisiae* from the same source to test whether our observations from the human proteome generalized to other species.

The human proteome download contains predictions for some very long protein sequences, which were broken into overlapping fragments for prediction. Predictions treated in this fashion were useful in the early stages of our survey, as they provided examples with especially high *barbed wire* content (Fig. 2). Fragmented predictions were excluded from our final analyses, as the fragmented sequences lacked their full context, the predictions appeared to be worse than for nonfragmented sequences, and the proper way to count the overlapping regions of the fragments was unclear.

### 2.2. Structure survey

We surveyed the human proteome predictions, focusing on low-pLDDT regions. The initial survey was visual, using *MolProbity* validation markup and all-atom contacts in our *KiNG* software (Chen *et al.*, 2009 ▸) to identify patterns of behavior.

We developed an analysis tool (described below) to automate our visual observations, categorizing *AlphaFold*2 residues into behavioral modes based on pLDDT, packing and validation criteria. The tool can output text or JSON annotations of residues, a structure file pruned to include only residues of selected modes, or visual annotations in the form of kinemage markup viewable in *KiNG*.

### 2.3. MobiDB survey

For each *AlphaFold*2 structure we categorized, we downloaded the corresponding MobiDB entry based on UniProt ID in JSON format. Each MobiDB entry provides ranges of residues from that sequence that fit within a variety of disorder categories. For each of our categorized residues, for each MobiDB disorder category, we determined whether or not the residue was within the MobiDB residue range, and added up the totals. Results are presented as the fraction of our categorized residues that share each MobiBD disorder annotation.

### 2.4. *phenix.barbed_wire_analysis* tool

The tool accepts a structure file in PDB or mmCIF format, with pLDDT in the *B*-factor field, as per the *AlphaFold* standard. Hydrogens are added to a submitted structure with *Reduce*, and contact analysis is performed with *Probe* (Davis *et al.*, 2007 ▸). Secondary-structure elements are identified based on CA geometry. For each residue, a packing score is determined based on the number of different steric contacts (0.5 Å van der Waals surface separation or closer) per non-H atom in a five-residue window (*i* −2 to *i* + 2) around the residue of interest. Where secondary structure is identified, contacts internal to a given secondary-structure element (such as the hydrogen bonds between β-strands) are not counted. Likewise, local contacts within a sequence distance of 4 are omitted from the count. The purpose of these omissions is to focus on contacts from tertiary structure and ensure that helices and sheets must touch other elements of the structure to count as packed, despite their rich internal contacts. For helix and coil residues, a score of >0.6 contacts per heavy atom is considered adequately packed. For β-strand residues, whose packing may be dominated by (omitted) intra-sheet contacts, the cutoff score is lowered to 0.35.

*MolProbity* validations are run via *Phenix* (Liebschner *et al.*, 2019 ▸), namely *ramalyze* for Ramachandran (Lovell *et al.*, 2003 ▸; Ramachandran *et al.*, 1963 ▸), *CaBLAM* (Williams *et al.*, 2018 ▸), *omegalyze* for *cis* and twisted peptide bonds (Williams *et al.*, 2018 ▸) and *mp_validate_bonds* for covalent bond geometry (Moriarty *et al.*, 2014 ▸).

A residue is marked as having a high density of outliers if two or more of the following are true about a three-residue window centered on that residue: two or more residues have *cis*-nonPro or twisted peptide bonds, two or more residues have *CaBLAM* and/or CA geometry outliers, two or more residues have covalent bond-length and/or bond-angle outliers, or all three residues fall in a high-ψ Ramachandran band (+60° < ψ < +170°) and at least one residue is a Ramachandran outlier.

An individual residue is marked as having a signature *barbed wire* outlier if it is a Ramachandran outlier falling in the upper right of the Ramachandran plot (−15° < φ < +170°, +60° < ψ < +170°). A residue is also marked as having a signature *barbed wire* outlier if it is a CA geometry outlier as determined by *CaBLAM* and is also in a low-pLDDT, unpacked region (this outlier is permitted in predictive regions). Since *barbed-wire*-ness appears to be a property of joining residues together, both residues that share a peptide bond are marked as having a signature outlier if that peptide bond contains a C—N—CA bond-angle outlier, a *cis*-nonPro peptide bond or any twisted peptide bond. A *cis*-Pro is permitted in predictive regions and is only considered to be a signature outlier if it appears in a low-pLDDT and unpacked region.

**Figure 1:**
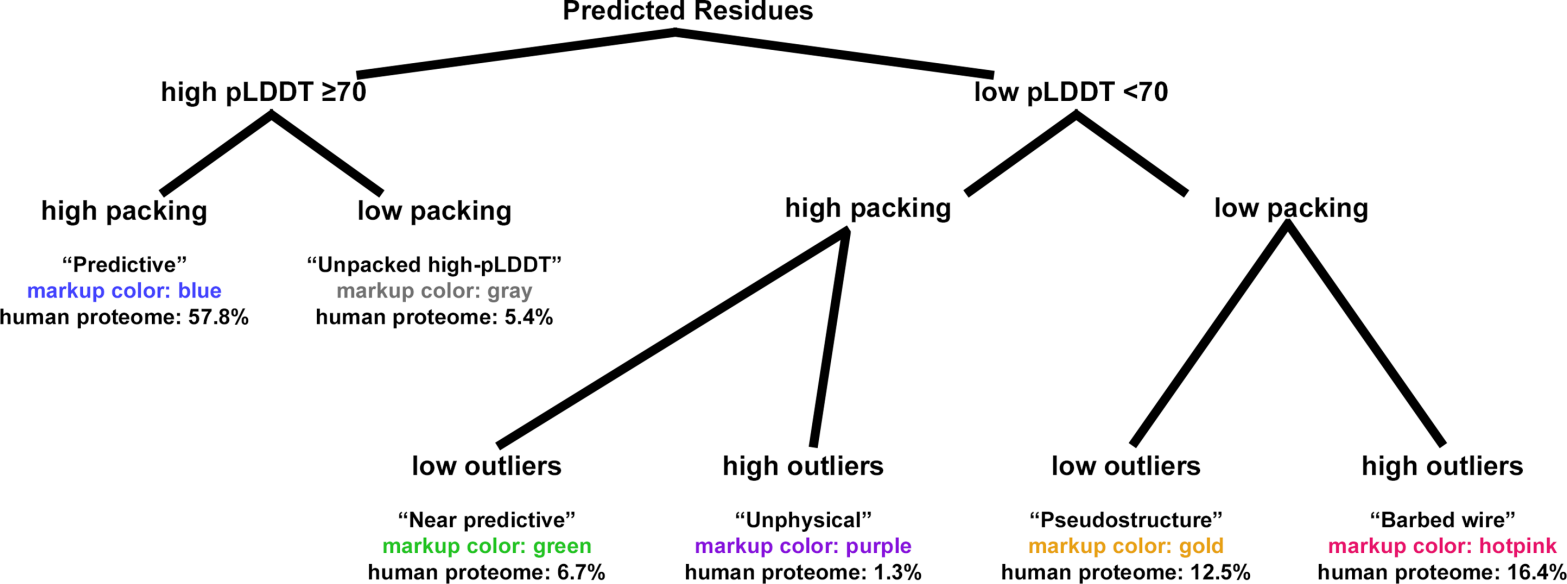
Prediction modes of *AlphaFold*2 and their relationships. This tree diagram shows how *AlphaFold*2 residues are divided into our modes, first by pLDDT, then by contact packing and then by validation outliers if necessary. Validation outliers are rare in high-pLDDT regions and are not used to define additional modes. For each mode, the name is listed, followed by the color used in our tool’s kinemage markup, followed by the frequency of that mode within the human proteome predictions. In the kinemage, markup for each mode can be toggled individually to aid readability. Example markup is shown in Fig. 4 ▸.

Residues are then categorized into our defined modes (Fig. 1 ▸). *Predictive* residues are high-pLDDT and high packing. *Unpacked high-pLDDT* residues are high-pLDDT but low packing. *Near-predictive* residues are low-pLDDT, high packing with low outliers. *Pseudostructure* residues are low-pLDDT, low packing with low outliers. *Barbed wire* residues are low-pLDDT, low packing and show high outlier density, a signature *barbed wire* outlier or both. *Unphysical* residues are the relatively rare low-pLDDT, high packing, signature outlier/high outliers case.

To reduce fragmentation and simplify the annotation, isolated residues are smoothed into their neighbors. This is performed in two passes. In the first pass, if one or two residues of *pseudostructure* are surrounded on both sides by *barbed wire* or by *near-predictive*, the *pseudostructure* residues are recategorized to match their surroundings. In the second pass, if one or two residues of *barbed wire* are still surrounded by *pseudostructure*, the *barbed wire* residues are recategorized to *pseudostructure*.

Kinemage markup can be generated with phenix.barbed_wire_analysis output.type=kin. Besides color-coded balls on each CA (see Fig. 1 ▸ for colors), the kinemage markup includes text-label annotations for each residue showing how similar to *barbed wire* it is: L for low pLDDT, p for low packing, r for Ramachandran outlier, o for omega outlier (peptide-bond dihedral), c for *CaBLAM* outlier and g for covalent geometry outlier. These labels always occur in the same order, Lprocg, and each is replaced with a dash if the residue is not like *barbed wire* in that regard. Thus, L----- is the typical label for *near-predictive* residues and Lp---- is the typical label for *pseudostructure*.

A pLDDT 70 cutoff suffices for most *AlphaFold* prediction preparation and is the default in *Phenix*’s *Process Predicted Model* and *CCP*4’s *Slice’n’Dice* (Simpkin *et al.*, 2025 ▸). In cases where significant regions of a prediction are below pLDDT 70, the barbed-wire analysis tool can provide an alternative preparation with phenix.barbed_wire_analysis output.type=selection_file, printing a PDB file that contains only residues from selected modes. By default, *predictive* and *near-predictive* residues are returned, but any combination of modes may be selected with the modes=# flag. However, in challenging cases, we recommend leveraging the ‘intelligence augmentation’ (Engelbart, 2023 ▸) of the complete kinemage markup rather than relying on automation.

## 3. Results

At low pLDDT, we observed three primary modes and one minor mode of *AlphaFold* prediction, all mutually exclusive, based on the combinations of high or low packing contacts with high or low density of validation outliers (Fig. 1 ▸). At one extreme is the *near-predictive* mode, which strongly resembles folded protein. At the other is *barbed wire*, which has essentially no protein-like properties. By applying packing criteria to high-pLDDT regions, we defined two additional prediction modes, *predictive* and *unpacked high-pLDDT*, for a total of six modes.

### 3.1. Barbed wire

**Figure 2:**
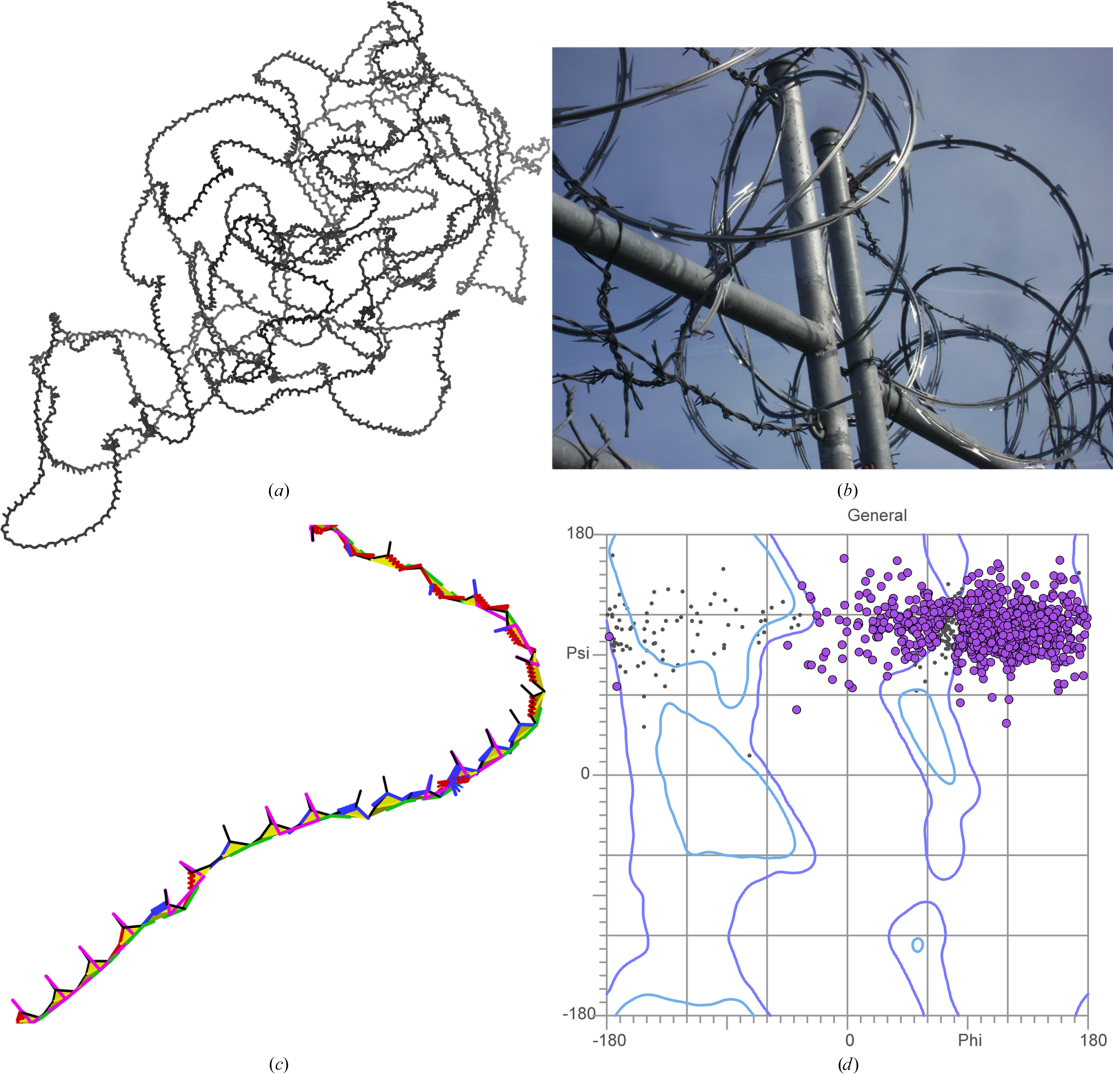
*Barbed wire* residues in *AlphaFold*2 predictions. (*a*) Nearly all-*barbed wire* prediction of fragment 6 of UniProt ID Q86YZ3 (very long sequences are predicted as multiple overlapping fragments). Wide, looping or tangled coils are typical of the barbed-wire prediction mode. (*b*) Real barbed wire, whose spikes and coils give the prediction mode its name. (Image credit: Smithers7 by way of Wikimedia Commons, Creative Commons Attribution 3.0 Unported). (*c*) Zoomed-in view of the Q86YZ3 fragment 6 prediction, residues 962–1005, with *MolProbity* validation markup, showing an extremely high density of validation outliers. Markup is green for Ramachandran outliers, red and blue for covalent geometry outliers, magenta for *CaBLAM*, lime green and yellow for *cis* and twisted peptide bonds. CA geometry outliers from *CaBLAM* are omitted for clarity but are pervasive. Carbonyl oxygen bonds are frequently pointed in the same direction, rather than alternating as in β-strands. (*d*) Ramachandran distribution for general-case residues in the Q86YZ3 fragment 6 prediction. Outliers are marked in purple. The distribution is highly unusual and clustered in the upper right of the plot, corresponding to an extended but unprotein-like conformation.

Many low-pLDDT regions are typified by wide, looping coils and by spike-like near-parallel arrangements of backbone carbonyl oxygens (Fig. 2 ▸*a*). To our eyes, these features are reminiscent of coils of barbed wire (Fig. 2 ▸*b*), so we have given them that name. ‘Spaghetti’ is a metaphor in common use for these regions (Jones & Thornton, 2022 ▸; Varadi *et al.*, 2024 ▸), but we do not favor the pasta analogy as it implies both flexibility (in contrast to the wide, rigid-looking arcs we observe) and closely piled packing (in contrast to the extremely low packing density we use to define this mode). Occasionally, these regions are referred to as ‘ribbon-like’ (Abramson *et al.*, 2024 ▸; Piovesan *et al.*, 2022 ▸), but we strongly disfavor that metaphor, given our historical connection to the development of ribbon diagrams for representation of protein α-helices and β-sheets (Richardson, 2000 ▸), which *barbed wire* emphatically does not resemble.

More diagnostic than these visual features is the extreme unprotein-likeness of *barbed wire* regions. *Barbed wire* residues are almost entirely unpacked; these great looping coils afford essentially no local steric contacts, and the coils generally do not pass near other parts of the structure. Additionally, *barbed wire* residues have an extremely high rate of backbone geometry outliers when assessed with *MolProbity* structure validation (Prisant *et al.*, 2020 ▸). We observed that each residue in a *barbed wire* region typically manifests at least two (and frequently more) of the following: Ramachandran outliers, *CaBLAM* outliers, *cis* or twisted peptide bonds, covalent bond-length outliers and covalent bond-angle outliers (Fig. 2 ▸*c*). Ramachandran outliers in *barbed wire* regions occur primarily in the upper right portion of the Ramachandran plot (Fig. 2 ▸*d*).

Cβ deviation outliers (Lovell *et al.*, 2003 ▸) are also common, although less pervasive or diagnostic. Surprisingly, given the density of other outliers, *barbed wire* residues show very few side-chain rotamer outliers or steric clashes.

**Figure 3:**
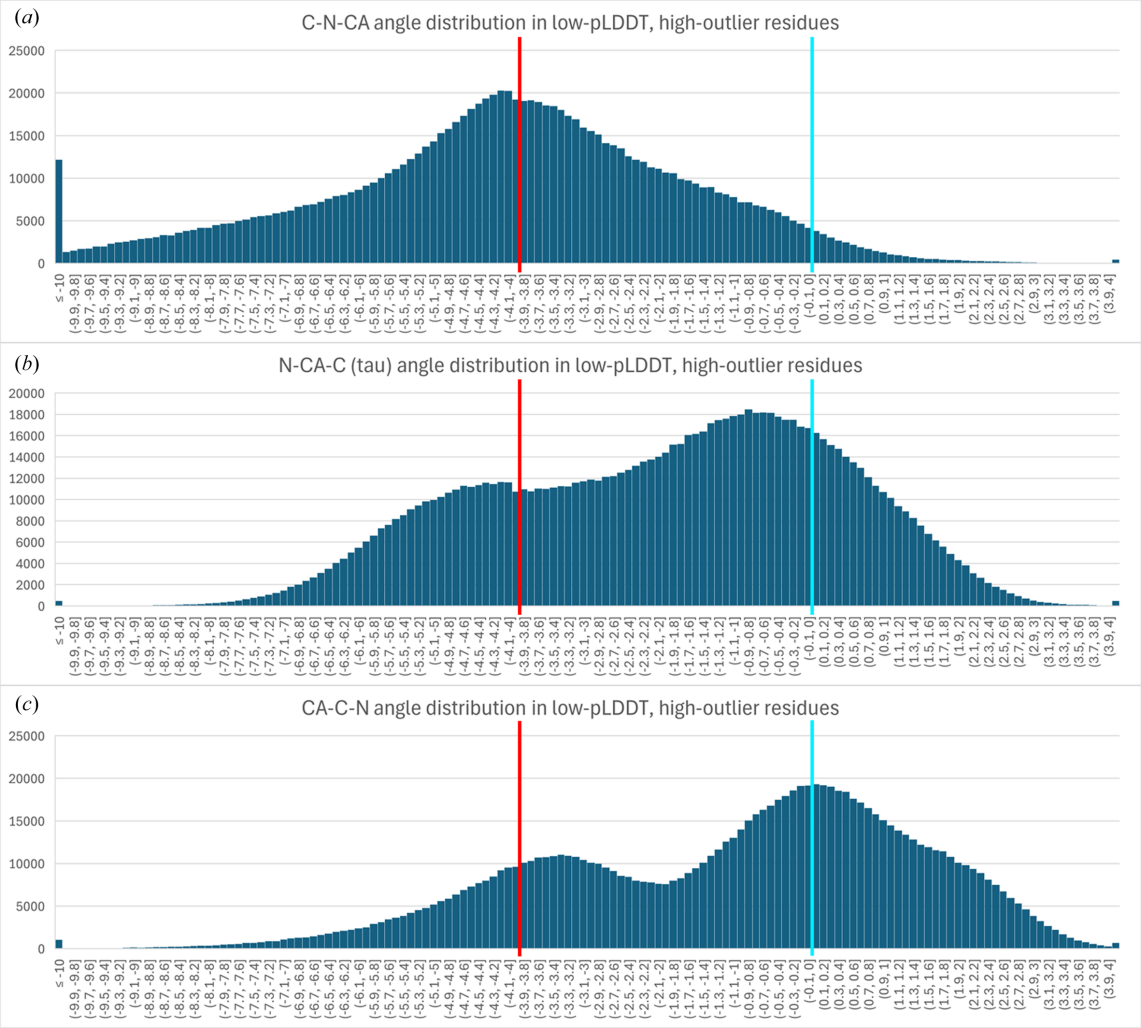
Histogram distributions of backbone covalent bond angles from *AlphaFold*2 prediction residues with low pLDDT, low packing and high outlier density (these residues are *barbed wire*-like, but without explicit selection for angle outliers). The *x* axis is σ from the target angle value, with bins of 0.1σ. The *y* axis is the count of residues falling in the bin. The target (0σ) is marked with a light blue bar; the outlier threshold (−4σ) is marked with a red bar. The underflow bin is ≤−10σ; the overflow bin is >+4σ (the other outlier threshold). All three of these bond angles show frequent geometric distortions, with the distortion of the C—N—CA angle being systematic, and its distribution recentered almost exactly on the −4σ outlier threshold (*a*). This angle partially spans the peptide bond, and its systematic distortion suggests errors in how *AlphaFold* forms peptide bonds in *barbed wire*.

Backbone covalent bond angles are of particular interest for diagnosing and understanding *barbed wire*. Although any of the backbone covalent bond angles within *barbed wire* may be distorted, we found that the C—N—CA bond angle is systematically abnormal in low-pLDDT regions with high outlier density (Fig. 3 ▸). The peak of the distribution for these C—N—CA angles falls at about −4σ, our typical cutoff for identifying bond-angle outliers.

It is likely possible to define a geometric description for our visual intuition that *barbed wire* occurs in wide, rigid coils. However, this additional description was not necessary at this time. We found that the combination of packing and validation was sufficiently diagnostic, especially since C—N—CA angle outliers, *cis* and twisted peptide bonds, and upper-right quadrant Ramachandran outliers serve as consistent signatures for the *barbed wire* mode. (See Section 2.4 for a complete description of how signature outliers are treated.)

Users evaluating *AlphaFold* predictions with *MolProbity*or other validation software must take care. The presence of *barbed wire* regions will greatly worsen the apparent quality of whole-model validation statistics (for example overall Ramachandran outlier percentage), without reflecting on the actual quality of the high-pLDDT or otherwise well predicted regions of that structure. Similarly, the predicted template–model (pTM) and interface predicted template–model (ipTM) scores of whole-model *AlphaFold* prediction accuracy perform better when a distinction is first made between ordered and disordered regions of the prediction (Dunbrack, 2025 ▸). As always with validation, local details and context matter more than whole-model averages.

### 3.2. Near-predictive

At the other extreme from the underpacked and unprotein-like *barbed wire* are *near-predictive* regions. These are regions of low-pLDDT prediction that nevertheless have protein-like packing and geometry. Some of these *near-predictive* regions are cases where *AlphaFold* has produced a mostly correct prediction, but the confidence score does not reflect that correctness. *Near-predictive* regions can have a few validation outliers and steric clashes, similar to high-confidence regions and experimental structures, but do not have a diagnostic pattern of outliers.

After identifying *near-predictive* regions of human *AlphaFold*2 predictions, we surveyed the PDB in search of experimentally solved versions of those same regions to assess the accuracy of the predictions. We identified 551 predictions containing a continuous *near-predictive* segment of at least 30 residues. Of these, 129 had a deposited structure in the PDB containing a chain with the same UniProt ID, and only 35 had an experimentally solved chain that covered a significant number of residues predicted at low pLDDT.

**Figure 4:**
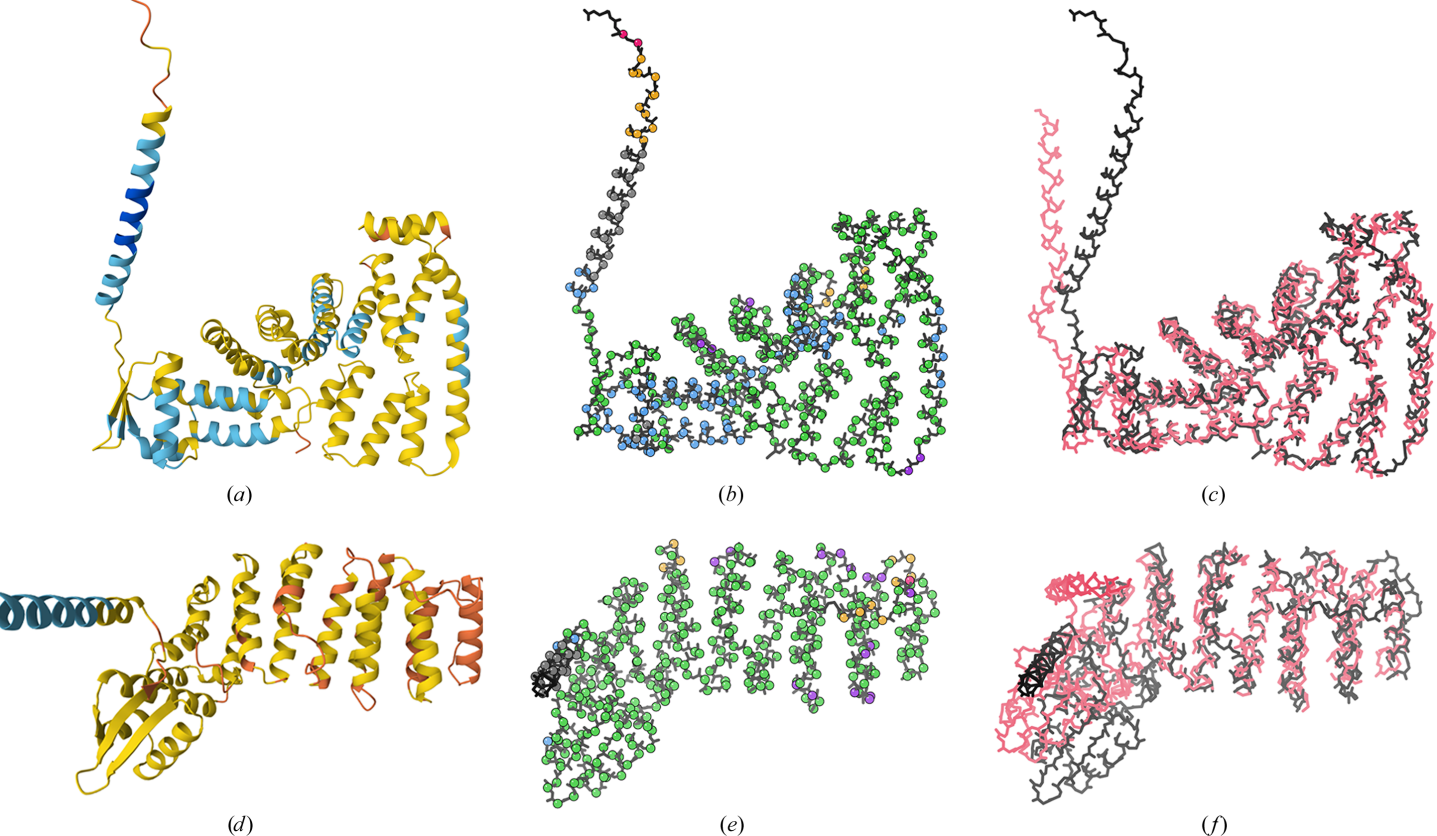
*Near-predictive* residues from *AlphaFold*2 predictions of eukaryotic translation initiation factor 3 and their experimentally solved counterparts. (*a*) Prediction of UniProt ID P60228 with standard pLDDT coloring from the Mol* viewer at the AlphaFold Database. (*b*) Prediction of UniProt ID P60228 with markup from our *barbed_wire_analysis* tool. The structure is primarily *predictive* (blue) and *near-predictive* (green), with the top-left helix an example of *unpacked high pLDDT* (gray). (*c*) Superposition of the prediction (black) with PDB entry 6zon chain *E* (pink), solved by cryoEM at 3.0 Å resolution. (*d*) Prediction of UniProt ID Q7L2H7 with standard pLDDT coloring. This prediction is lower confidence than (*a*). (*e*) Prediction of UniProt ID Q7L2H7 with markup from our *barbed_wire_analysis* tool. The structure is primarily *near-predictive* (green), with some *unphysical* (purple) regions. (*f*) Superposition of the prediction (black) with PDB entry 6zon chain *M* (pink). Even at low confidence, these predictions are close enough to the experimental structures to have structural meaning. The regions marked as *unphysical*, indicating severe validation outliers, correlate with loops omitted from the experimental structures.

Experimental versions of *near-predictive* regions solved prior to *AlphaFold*2 are rare. Sequences that fold stably and behave well experimentally appear strongly correlated with sequences that *AlphaFold*2 predicts with high confidence. The most dramatic examples of *near-predictiveAlphaFold* regions matching experimental structures are domains from eukaryotic translation initiation factor 3, including subunits *A*, *E*, *L* and *M*. The predictions of UniProt IDs P60228 (Fig. 4 ▸*a*) and Q7L2H7 (Fig. 4 ▸*d*) have low and very low pLDDT confidence scores, respectively, but nevertheless align very well with chains *E* and *M* of PDB entry 6zon, experimentally solved at 3.0 Å resolution by electron microscopy (Thoms *et al.*, 2020 ▸). There are some distortions, especially in or near the *unphysical* loops marked with purple spheres in Fig. 4 ▸(*e*), which is part of why we designate this mode as *near-predictive* rather than fully predictive. However, these predictions are clearly near enough to the target to be useful for many structural biology purposes, especially if supplemented with experimental data as in *Phenix*’s iterative *Predict and Build* pipeline (Terwilliger *et al.*, 2023 ▸). We also found good matches between near-predictive regions and experimental structures for PDB entry 3LLu chain *A* with UniProt ID Q9HB90 and PDB entry 6qkL chain *J* with UniProt ID Q9Y3A5.

Post-*AlphaFold*, experimental structures that used predictions in model building are increasingly common. PDB entries 8cr1, 8fw5, 9dtq and 9nh8 all used predictions with near-predictive regions and those regions proved to be good matches to the experimental maps. Of these, PDB entry 9dtqis particularly notable. Chains *B* and *E* of this structure are solutions of UniProt ID Q9NVX7. The only pre-*AlphaFold* structure for this UniProt sequence, PDB entry 2eqx, solves the relevant near-predictive region in a different conformation (probably due to being a fragment of the full sequence). These cases show that the inclusion of near-predictive regions in model building can indeed contribute to successful experimental models and that *AlphaFold*2 predictions are not necessarily dominated by PDB templates.

There is some indication that *AlphaFold*3 (Abramson *et al.*, 2024 ▸) more accurately assesses the confidence of regions that were *near-predictive* under *AlphaFold*2 and assigns them higher pLDDT scores. However, without a proteome-level resource of *AlphaFold*3 predictions, systematic study of *AlphaFold*3’s behavior in former and current low-pLDDT regions will be limited.

### 3.3. Pseudostructure

**Figure 5:**
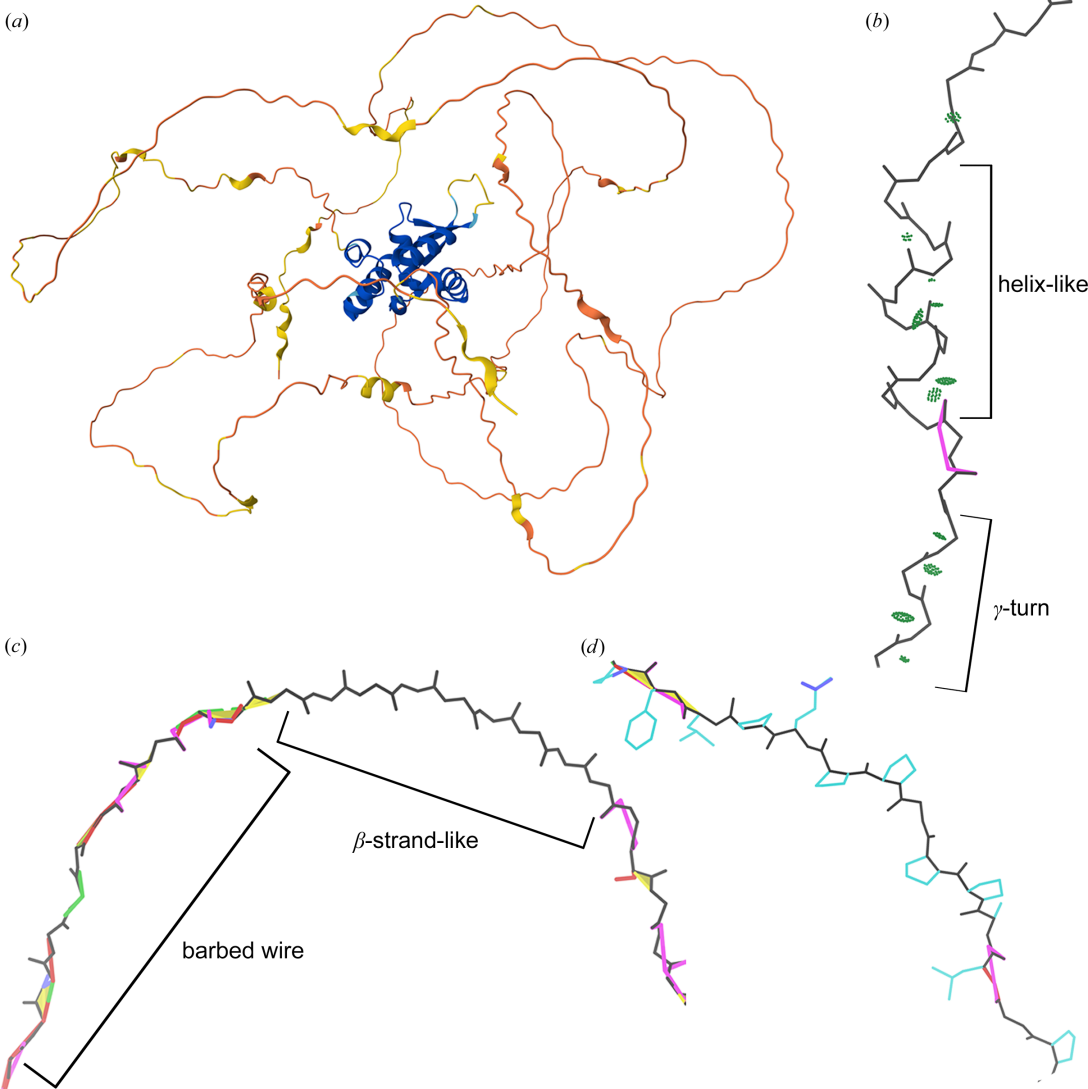
Examples of low-pLDDT *pseudostructure* predictions from UniProt ID O15353, human forkhead box protein N1. (*a*) Overall prediction colored by pLDDT from the Mol* viewer at the AlphaFold Database. A well packed and well predicted core is surrounded by *barbed wire* and *pseudostructure*. Several *pseudostructure* elements are sufficiently similar to secondary structure to be depicted as ribbons by Mol*. (*b*) A pseudostructure helix, residues 544–554. Hydrogen bonding (light green dots arranged in pillow shapes) is inconsistent or weak, and the helix is not well formed. A γ-turn-like segment, a rare *pseudostructure* feature, is visible before/below the helix. (*c*) A *pseudostructure* β-strand, residues 161–169. This strand is unpaired, so has no hydrogen bonding. *Barbed wire* regions before and after the strand show the sharp difference in validation outlier density, even though all of this strand is predicted at very low confidence (mostly pLDDT < 40). (*d*) A polyproline II region, residues 464–470. This conformation is correctly associated with regions of high proline content in *AlphaFold* predictions, but often occurs with low pLDDT.

Between *barbed wire* and *near-predictive*, we observe a third mode of behavior, having protein-like residue geometry but minimal packing contacts. In addition to having legal peptide-bond arrangements, *pseudostructure* residues generally assume conformations that are sufficiently similar to recognizable secondary-structure elements (Wilson *et al.*, 2022 ▸) that the Mol* viewer (Sehnal *et al.*, 2021 ▸) at the AlphaFold Protein Structure Database draws many of them as ribbons instead of coil (Fig. 5 ▸*a*). However, on closer inspection, *pseudostructure* regions generally lack features of well formed structure.

*Pseudostructure* helices are often stretched or extended, and they have severely weakened or absent *i* to *i* + 4 hydrogen bonding (Fig. 5 ▸*b*). *Pseudostructure* β-strands similarly lack hydrogen bonding, being isolated and unpaired (Fig. 5 ▸*c*).

*Pseudostructure* also contains polyproline II-like regions (Fig. 5 ▸*d*). Polyproline II (Adzhubei *et al.*, 2013 ▸; Sasisekharan, 1959 ▸) is a repeating conformation formed by successive prolines with Ramachandran conformation around φ = −75°/ψ = 150°. Regions that *AlphaFold*2 predicts as polyproline-like in conformation are indeed usually polyproline-like in sequence, having a very high percentage of proline residues. These polyproline regions may extend for more residues than polyproline typically does in ordered regions of experimental structures, but are otherwise among the most consistently well formed of the *pseudostructures*.

We also observe occasional regions of extended γ-turn conformation (Matthews, 1972 ▸), with an *i* to *i* + 2 hydrogen-bonding pattern (Fig. 5 ▸*b*). This is a rare *pseudostructure* conformation and one of the only ways we observed to generate a consistent hydrogen-bonding pattern at low pLDDT.

### 3.4. Unphysical

We find that high-pLDDT, predictive regions of *AlphaFold*2 structures are overall similar to well solved experimental structures in *MolProbity* validation quality. Validation outliers occur at a reasonable rate (*i.e.* rarely, but not never) and are generally not diagnostic of some underlying prediction phenomenon. This behavior extends to *near-predictive* regions, which likewise generally conform to validation expectations.

Regions with high rates of both validation outliers and packing, designated *unphysical*, are therefore surprising. While *barbed wire* loops are generally well separated from other structure in space, they occasionally pass close enough to other structure to appear packed. The most drastic form of this close approach is an actual intersection between parts of the chain, which results in strong steric clashes in addition to other severe validation outliers. Chain intersections were the only case where we systematically observed severe steric clashes at low pLDDT.

While most of these regions are properly understood as a variation on *barbed wire*, we give high-outlier high-packing regions the separate *unphysical* designation to draw attention to their association with impossible chain intersections.

Recognition of the *unphysical* mode is an advancement over *AlphaCutter* (Tam & Iwasaki, 2023 ▸). In our testing with its default settings, *AlphaCutter* tends to accept chain intersections and close approaches as valid globular-like structure due to their high contact packing and despite their physical impossibility.

An interesting case of *unphysical* residues can be seen in the Q7L2H7 eukaryotic translation initiation factor 3 subunit *M* prediction (Fig. 4 ▸*e*). The residues identified as *unphysical* (purple spheres) are largely at or near loops that have been omitted from the PDB entry 6zon reference structure (Fig. 4 ▸*f*, pink backbone). These loops are short, and in the predicted model they do not extend far enough from the rest of the structure to lose packing. Validation outliers in the predicted model thus correspond to regions of greater flexibility or uncertainty in the experimental structure, and the *unphysical* designation marks residues with low predictive value.

### 3.5. High-pLDDT prediction modes

Contact analysis also elucidates an additional minor prediction mode at high pLDDT. Besides the main *predictive* mode, which is protein-like in its high degree of contact packing, there are underpacked regions that are nevertheless predicted at high or very high confidence. We call these *unpacked high-pLDDT*. *Unpacked high-pLDDT* regions are most often long helices that stick out from the well predicted core of a structure (Fig. 4 ▸*a*). These regions will generally be explicable in the context of the structure; for example, many are conditionally ordered (Alderson *et al.*, 2023 ▸), or are membrane or domain insertion helices. All of these cases may need to be trimmed from an experimental construct to facilitate crystallization.

### 3.6. pLDDT and prediction modes

**Figure 6:**
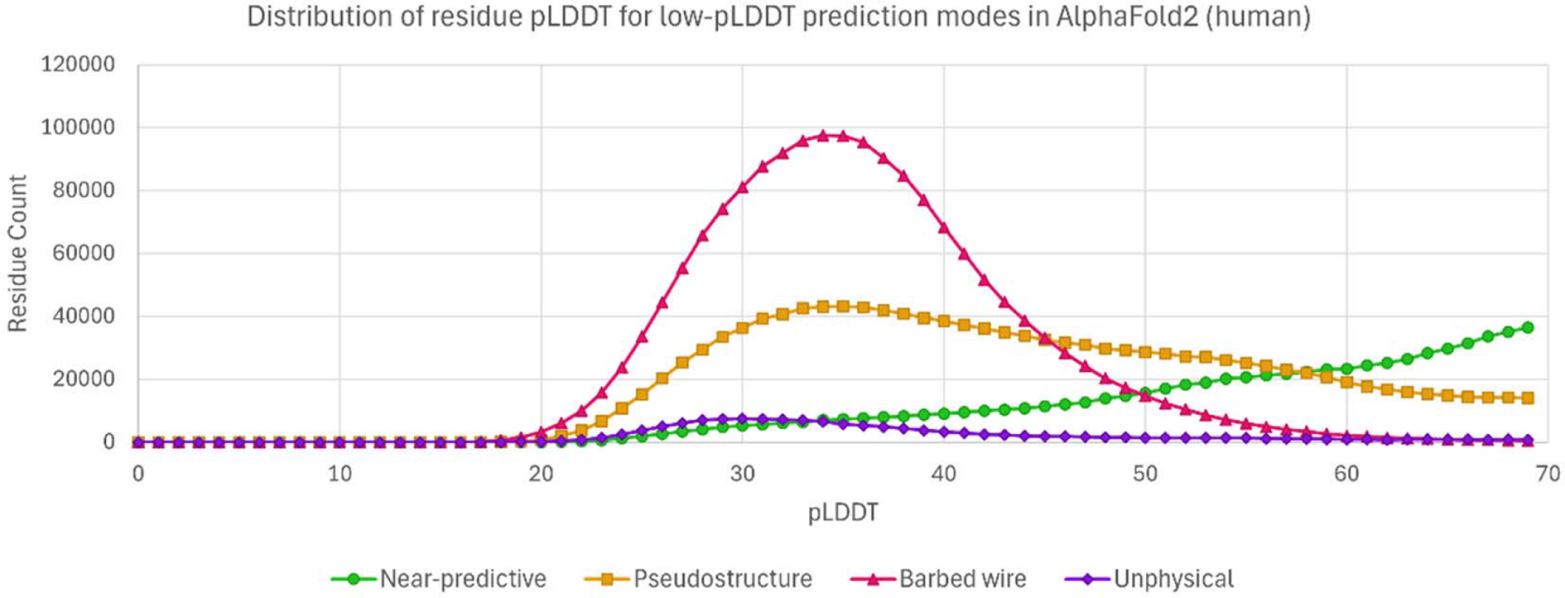
pLDDT distibutions for the major low-pLDDT prediction modes, with pLDDT bins of 1, for sequences from the human proteome. *Barbed wire* (red, triangles) correlates with lower pLDDT scores. *Near-predictive* (green, circles) correlates with higher pLDDT scores. The crossing point for *barbed wire* and *near-predictive* is close to 50; the yellow/orange boundary in conventional pLDDT coloring. If not for *pseudostructure* (gold, squares), which correlates only weakly with low pLDDT, a pLDDT 50 cutoff would be sufficient to select for most *near-predictive*.

We determined the pLDDT distributions of residues from the low-pLDDT prediction modes (Fig. 6 ▸). pLDDT has a minimum observed value around 20. *Barbed wire* shows a strong and almost diagnostic preference for very low pLDDT. *Unphysical* is rare, but occurs mostly at very low pLDDT, like *barbed wire. Near-predictive* prefers higher pLDDT. *Pseudo­structure* populates the full low-pLDDT range from 20 to 70, with some preference for very low pLDDT. The lines for *barbed wire* and *near-predictive* cross near pLDDT 50, which corresponds to the yellow to orange transition in conventional *AlphaFold* pLDDT coloring (Figs. 4 ▸*d* and 5 ▸*a*). Most *barbed wire* residues can be avoided with a pLDDT 50 cutoff, but *pseudostructure* cannot be distinguished from the other modes by pLDDT alone and requires other analyses.

The high and defined *barbed wire* peak supports our interpretation that *barbed wire* represents a distinct prediction behavior. The broad and weakly multi-modal distributions of *pseudostructure* and *near-predictive* suggest that these modes each contain multiple behaviors.

The pLDDT distribution (Supplementary Fig. S21) for *S. cerevisiae* is similar to human. pLDDT distributions for the prokaryotes *E. coli* (Supplementary Fig. S23) and *S. aureus* (Supplementary Fig. S23) show much lower peaks for barbed wire, especially in *E. coli*. The barbed wire peak for *E. coli* is also shifted towards higher pLDDT, being centered at about pLDDT 40, while the peaks for other species are centered at about pLDDT 35. This is likely due to prokaryotes having fewer intrinsically disordered regions than eukaryotes and *E. coli* being a particularly well studied organism with an especially rich multiple sequence alignment (MSA) and templates for *AlphaFold* to draw from.

### 3.7. Sequence properties of prediction modes

The major prediction modes at low pLDDT, *barbed wire*, *pseudostructure* and *near-predictive*, exhibit clearly different physical behaviors. We sought an explanation for why *AlphaFold* produces these different behaviors. In particular, is there a distinction between *barbed wire* and *pseudostructure* that might elucidate some property of disordered or partially ordered regions?

The MobiDB database (Piovesan *et al.*, 2025 ▸) collects and presents many annotations and predictions of disorder. We surveyed regions of *barbed wire*, *pseudostructure*, *near-predictive* and high-pLDDT *AlphaFold*2 prediction modes for correlations with MobiDB annotations. To reduce the effects of edge definitions and smoothing, we only considered residues from uninterrupted (pre-smoothing) segments of *barbed wire*, *pseudostructure* and *near-predictive* that were at least three residues long after the first and last three residues were removed from the segment. This aggressive pruning focused the survey on only unambiguous cases of each prediction mode. High-pLDDT regions were identified with a simple pLDDT ≥ 70 cutoff and include both our *predictive* and *unpacked high-pLDDT modes*.

**Figure 7:**
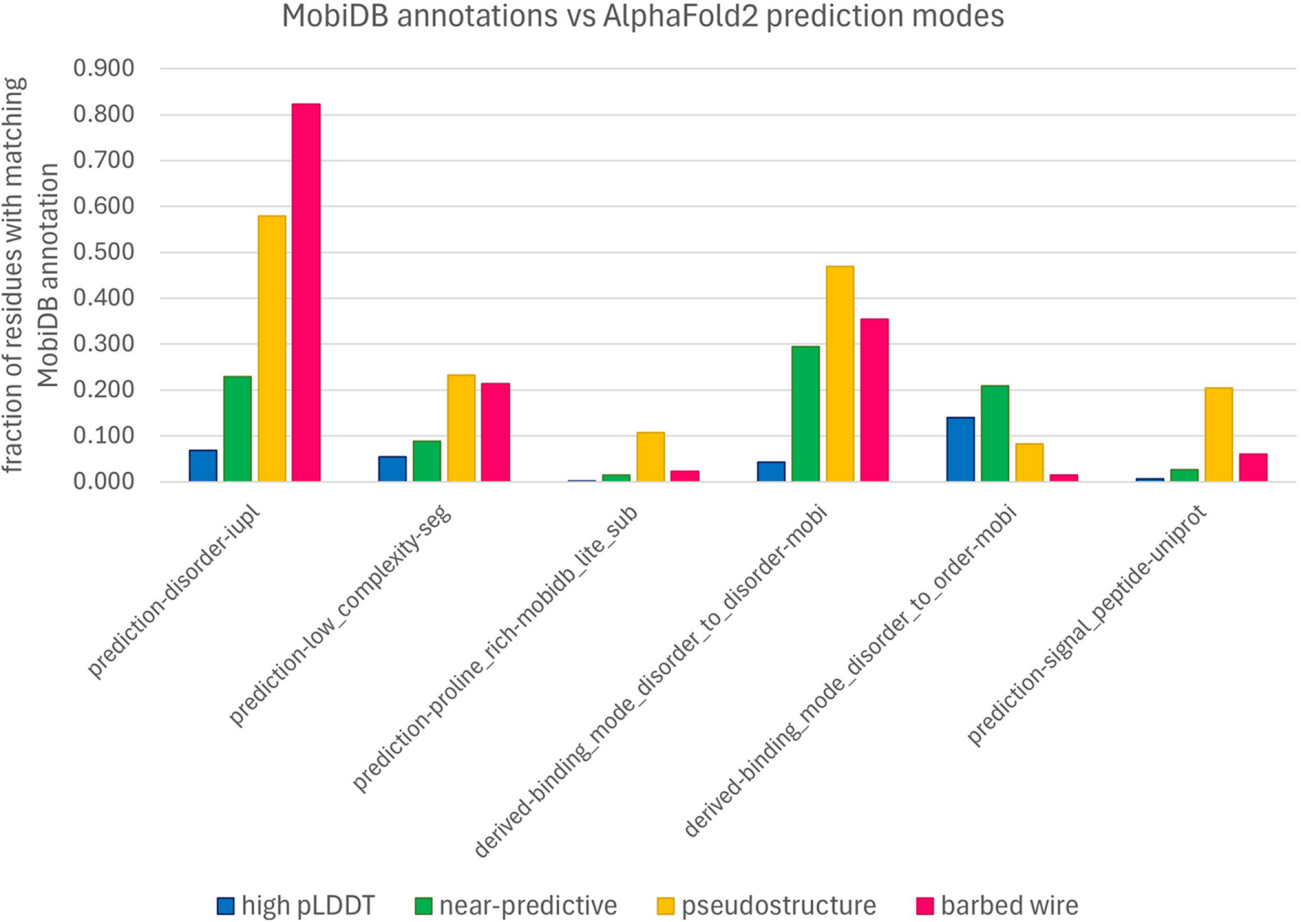
Relationships of *AlphaFold*2 prediction modes with MobiDB disorder annotations. From left to right: prediction-disorder-iupl shows a typical distribution for many predictions of disorder (see supporting information), where high-pLDDT residues are least associated with disorder and *barbed wire* residues are most associated, but no mode stands out as exceptional. The distributions to the right are exceptions to this general pattern. Low-complexity sequences are about equally associated with both *pseudostructure* and *barbed wire*. Proline-rich sequences are preferentially associated with *pseudostructure*, where we observe the polyproline II conformation. Disorder-to-disorder binding somewhat favors *pseudostructure*. Disorder-to-order binding somewhat favors *near-predictive*. Predicted signal peptides strongly favor *pseudostructure* over any other mode.

The complete MobiDB frequency analysis is provided in the supporting information; Fig. 7 ▸ excerpts points of interest from the complete analysis. Many of the MobiDB annotations show a stair-step pattern, with high-pLDDT residues the least correlated with disorder annotations, then *near-predictive*and then *pseudostructure*, with *barbed wire* being the most correlated with disorder (Fig. 7 ▸, prediction-disorder-iupl). This general pattern suggests that the difference between *barbed wire* and *pseudostructure* is one of degree rather than type.

However, certain annotations break from this pattern. We had hoped that low-complexity sequences would explain our prediction modes. The prediction-low_complexity-seg annotation indeed shows higher preference for the nonpredictive modes than for either of the predictive modes, but unfortunately does not distinguish between the nonpredictive modes. In retrospect, we recognize that low-complexity sequences are diverse in behavior and are not monolithically associated with either order or disorder. For example, prediction-proline_rich-mobidb_lite_sub shows a strong preference for the *pseudo­structure* mode over any other, in agreement with our observation that polyproline II conformations are regularly identified as *pseudostructure* in our system.

Derived-binding_mode_disorder_to_disorder-mobi shows some preference for *pseudostructure* over the other low-pLDDT modes. Derived-binding_mode_disorder_to_order-mobi shows a preference for *near-predictive* and a strong preference against *barbed wire*. The low pLDDT of *near-predictive* regions may therefore reflect a correlation with regions of conditional rather than permanent order. These results suggest that regions that *AlphaFold*2 predicts as *barbed wire* are not correlated with even conditional order. Annotations from *IDEAL* (Fukuchi *et al.*, 2012 ▸), a method focused on regions of conditional order, show similar patterns (see Supplementary Figs. S4 and S5). The most interesting preference revealed by the MobiDB survey is prediction-signal_peptide-uniprot. This annotation shows strong preference for *pseudostructure* above any other mode. This may represent a case where *AlphaFold*2 can recognize, by chance or design, a specific sequence property relevant to structural biology outside of folded domains.

These observations generalize to other examined proteomes: the eukaryote *S. cerevisiae* (see Supplementary Figs. S6–S10) and the prokaryotes *E. coli* (Supplementary Figs. S11–S16) and *S. aureus* (Supplementary Figs. S17–S20). In particular, signal peptides maintain a strong association with the *pseudostructure* mode across these organisms, although in *E. coli* they also appear in *barbed wire* regions.

## 4. Discussion

We identify several mutually exclusive modes of *AlphaFold*2 prediction behavior based on combinations of structure validation and packing criteria (Fig. 1 ▸). These are *predictive* (high pLDDT, high packing), *unpacked high-pLDDT* (high pLDDT, low packing), *near-predictive* (low pLDDT, high packing, low outliers), *pseudostructure* (low pLDDT, low packing, low outliers), *barbed wire* (low pLDDT, low packing, high outliers) and the relatively rare *unphysical* (low pLDDT, high packing, high outliers).

While a simple pLDDT ≥ 70 cutoff is sufficient for selecting the good parts of a prediction in most cases, understanding these more detailed prediction modes can be valuable for properly using a prediction. For example, the *unpacked high-pLDDT* mode often contains insertion helices which may need to be truncated from a construct to achieve crystallization. More positively, the *near-predictive* mode contains residues which, even at low confidence, may be closely related to the coordinates of the real structure. *Near-predictive* residues would be prime candidates for an iterative prediction process such as *Phenix*’s *Predict and Build*.

Contact packing is the single most important criterion for identifying *near-predictive* regions at low pLDDT, since serious validation outliers within well packed regions are rare. However, adding *MolProbity* validation criteria allows our *unphysical* category to annotate both residues of significantly worse prediction in otherwise *near-predictive* regions (see Fig. 4 ▸*e*) and sites of chain intersections, both of which can appear highly packed despite their errors. Validation criteria also introduce a distinction between *barbed wire* and *pseudostructure*.

### 4.1. Origins of prediction modes

Application of *MolProbity* validation reveals an apparent distinction between the *barbed wire* mode and the *pseudo­structure* mode based on *barbed wire*’s overwhelming density of backbone geometry errors. This is supported by *pseudo­structure*’s resemblance to secondary-structure elements compared with *barbed wire*’s completely unprotein-like conformation. A barbed wire segment is only a sequence-character prediction of disorder; the coordinate prediction should not even be treated as a member of an ensemble, as its extensive geometry outliers render its conformation physically impossible.

We speculate that the typical *barbed wire* conformation and its attendant validation outliers are not the result of *AlphaFold* prediction, but rather the lack of a prediction. These are probably regions that are effectively unpredicted, where the residues are strung together according to a default behavior to fill the necessary sequence space between predictable regions. This speculation that *barbed wire* represents a default behavior is supported by the strong association of validation outliers with the peptide bond. The commonness of C—N—CA bond-angle outliers and *cis* and twisted peptide bonds indicates a consistently unprotein-like assembly of adjacent residues. The unusual distribution of *barbed wire* residues in Ramachandran space also suggests a default behavior. As shown in Fig. 2 ▸(*d*), *barbed wire* residues are typically distributed in a band at high ψ (+60° to +170°) with a weaker preference for high φ (roughly −15° to +170°). This implies that the starting value of the ‘residue gas’ from which *AlphaFold* predictions are constructed, or possibly the default value after an initial residue movement, is at ψ ≃ +110° (ψ is the only Ramachandran angle specified for a single nonproline residue, since the O_*i*_ position also defines the N_*i*+1_ position across the planar peptide bond), and that the deviations from that value are needed to prevent clashes.

It is plausible that barbed wire represents regions that were abandoned at a relatively early stage of the prediction process. If this speculation is correct, then the high frequency of validation outliers in *barbed wire* regions may be considered a positive feature of such *AlphaFold* predictions, rather than a failure. The combination of low pLDDT and high outliers clearly marks *barbed wire* as distinct from regions where *AlphaFold* has completed its process, most importantly *near-predictive*.

If *barbed wire* is an arbitrary and abandoned assembly, then *pseudostructure* may be a partial prediction. The absence of validation outliers in *pseudostructure* suggests a more complete process.

However, it is also possible that *AlphaFold*2 has multiple patterns of arbitrary assembly and that *pseudostructure* represents patterns that happen not to generate *barbed wire*’s signature outliers. If this is the case, then *pseudostructure* is not fundamentally different from *barbed wire*, and is likewise an abandoned prediction. Our current analysis cannot entirely resolve the question of how, or whether, *pseudostructure* differs from *barbed wire*.

Polyproline II presents a particularly ambiguous case. We observed above that the ψ Ramachandran dihedral is defined by the geometry within a residue and that therefore the distinctive Ramachandran distribution of *barbed wire* residues (Fig. 2 ▸*d*) may reflect a default conformation within *AlphaFold*’s residue gas. In prolines, the φ dihedral is also heavily restricted by the side-chain geometry. The resulting φ, ψ combination would put a proline-rich residue gas in the correct Ramachandran region for polyproline II. Thus, at least for proline, even an arbitrary assembly process might generate the geometrically legal and sequentially correct polyproline II conformations we observe.

### 4.2. Sequence properties of prediction modes

Is there a sequence property that explains why *AlphaFold* produces such different-looking predictions in *pseudostructure* regions versus *barbed wire*? And if so, is that property relevant to structural biology, for example through correlation with intrinsically disordered regions (IDRs)? Our survey of disorder annotations from MobiDB was inconclusive. Broadly speaking, *near-predictive* regions had the most ordered-like residues at low pLDDT, according to MobiDB, and were substantially more ordered-like than *pseudostructure*. However, *pseudostructure* was only somewhat more ordered-like than *barbed wire* overall. Despite the association of signal peptides with *pseudostructure*, no MobiDB annotation category offered a clear explanation for why *AlphaFold* predicts some residues as *barbed wire* and others as *pseudostructure*. Nevertheless, we hope that this tool will be of use to others with a more nuanced understanding of disordered proteins.

*AlphaFold*2 is heavily dependent on multiple sequence alignment (MSA) for generating predictions. It is therefore possible that MSA depth could explain the *barbed wire*/*pseudostructure* distinction. The AlphaFold Protein Structure Database data sets do not report MSA depth information, so a complete analysis is not practical. However, MobiDB reports MSA occupancy for some sequences.

MSA occupancy is a per-residue score denoting the fraction of MSA sequences that contain a value for that residue. A high occupancy fraction indicates that most of the aligned sequences include that residue position. Only about 13% of the human proteome sequences, 7% of those from *S. cerevisiae* and 3.5% of those from *E. coli* from our study have MobiDB homology-msa_occupancy-psiblast annotations. Our analysis of MSA occupancy is therefore limited, especially since it is likely that the sequences which received homology-msa_occupancy-psiblast annotations are not independent of other sequence properties that might affect *AlphaFold* prediction.

**Figure 8:**
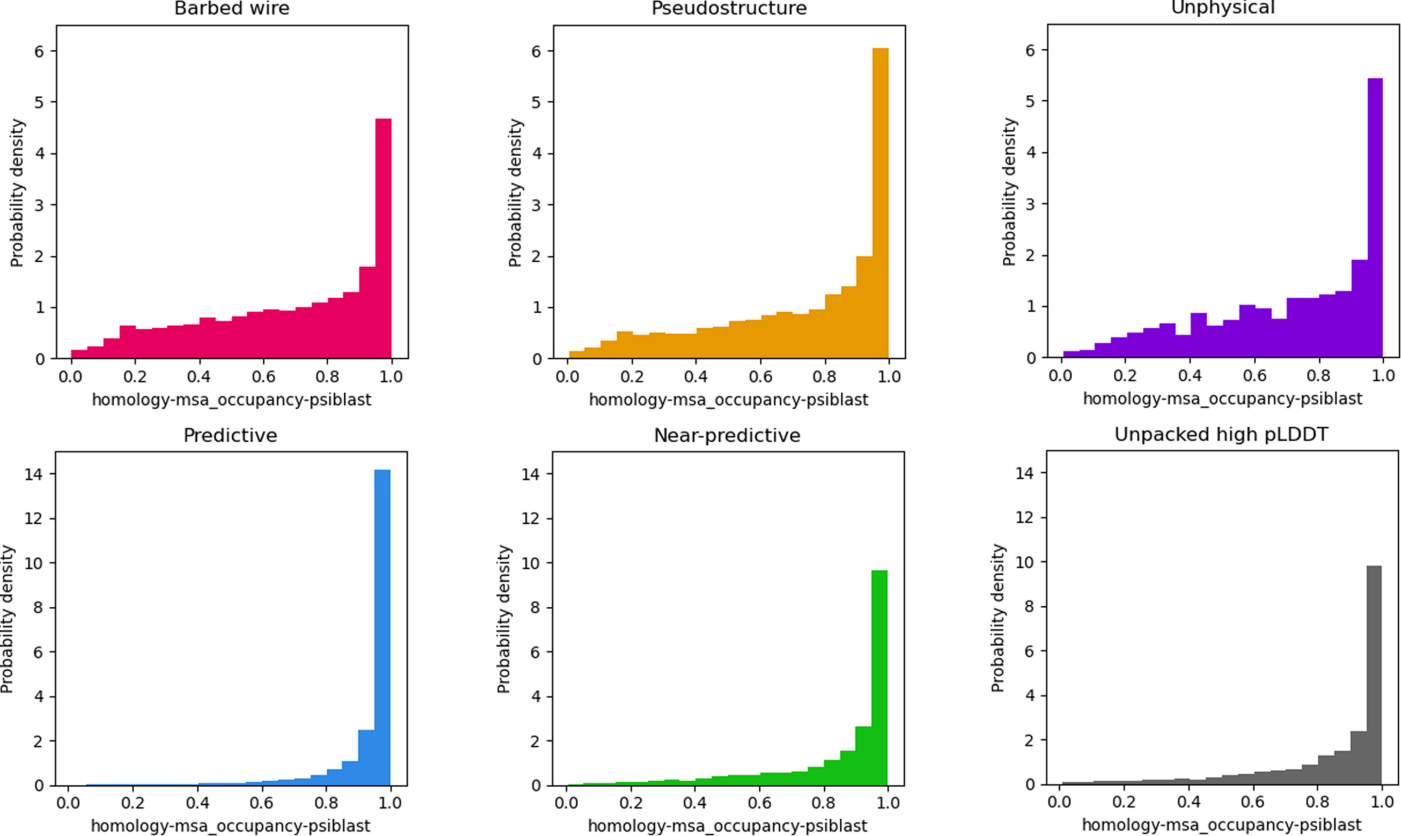
Multiple sequence alignment occupancy histogram distributions for *AlphaFold*2 human proteome predictions, as annotated in MobiDB. The three non-predictive modes (*barbed wire*, *pseudostructure* and *unphysical*) show similar distributions to each other. *Near-predictive* has a distribution more similar to the high-pLDDT modes, supporting our association of *near-predictive* regions with *predictive*. Similarity between *near-predictive* and *unpacked high-pLDDT* suggests that *near-predictive* also contains conditionally binding regions.

Fig. 8 ▸ shows probability density histograms for our six *AlphaFold*2 prediction modes. The nonpredictive modes, *barbed wire*, *pseudostructure* and *unphysical*, show similar distributions to each other. *Barbed wire* has a lower high-occupancy peak relative to *pseudostructure* and a higher distribution across the low-occupancy range. As with many of the other MobiDB annotations discussed above, *barbed wire* shows somewhat greater correlation to disorder than *pseudostructure*, but not enough to provide a clear explanation.

*Near-predictive* has a distribution much more similar to the high-pLDDT modes (*predictive* and *unpacked high-pLDDT*) than to the other low-pLDDT modes. This similarity supports our assumption that *near-predictive* regions are generally near-correct predictions – they have an MSA quality similar to *predictive* regions – but also reveals a more complex relationship with conditional folding.

MSA occupancy distributions from other organisms (see Supplementary Figs. S24 and S25) vary somewhat by species, with *E. coli* having a low peak across the middle range of occupancies and *S. cerevisiae* having a strong peak at very low occupancy. Despite these differences, the overall groupings are the same, with *barbed wire*, *pseudostructure* and *unphysical* similar to each other within each species, and *predictive*, *near-predictive* and *unpacked high-pLDDT* similar to each other.

### 4.3. Conditional folding

Work by Alderson *et al.* (2023 ▸), which focuses on relationships between intrinsic disorder and high-pLDDT regions, provides a useful companion piece to our work and is recommended. In particular, Alderson and coworkers explain many of the helices we identify as *unpacked high pLDDT*as regions of conditional order. They show that IDRs with high-pLDDT (pLDDT ≥ 70) *AlphaFold*2 predictions have favorable MSA properties (high alignment depth and high conservation) relative to IDRs with low-pLDDT (pLDDT < 50) predictions. However, they omit pLDDT between 50 and 70. Our tool can separate this difficult pLDDT range into major components of *near-predictive* and *pseudostructure* (Fig. 6 ▸). We find that *pseudostructure* residues have unfavorable MSA quality similar to *barbed wire* (Fig. 8 ▸), and therefore similar to residues predicted with pLDDT < 50. We find that *near-predictive* residues have favorable MSA quality similar to *unpacked high-pLDDT* residues, and therefore similar to the category of conditionally folding residues reported by Alderson and coworkers. Our survey of MobiDB annotations also found that disorder-to-order binding was associated with high-pLDDT and *near-predictive* regions, but not with *pseudostructure* or *barbed wire* (Fig. 7 ▸).

Taken together, these observations suggest that conditionally folded regions are an important component of *AlphaFold*2 predictions in the pLDDT 50–70 range, and that these regions are part of our near-predictive category. If *near-predictive* is populated by conditional folding (rather than consistent folding), that would help explain why experimentally solved versions of *near-predictive* regions were difficult to find in the PDB. The packing-based *near-predictive* versus *pseudostructure* distinction is especially important for identifying potential conditional folding, since *barbed wire* can be largely removed via a pLDDT cutoff.

However, as the eukaryotic translation initiation factor 3 examples illustrate (Fig. 4 ▸), the *near-predictive* mode is not exclusively conditionally folding. Much as the broader *pseudostructure* mode includes several distinct behaviors, *near-predictive* is not monolithic. Further investigation is needed to identify and interpret the most useful behaviors within this mode, and to understand how prediction of these regions changes from *AlphaFold*2 to *AlphaFold*3.

### 4.4. On templates

An important question, especially regarding *near-predictive* regions, is how much template structures from the PDB affect *AlphaFold*’s predictions. As with many questions about machine-learning methods, a clear answer is difficult to extract. Anecdotally, it seems that where a good multiple-sequence alignment exists the MSA dominates in *AlphaFold*2, and the presence or absence of templates matters less. However, it is also known that templates can be used to add positive information into *AlphaFold* during iterative prediction (Terwilliger *et al.*, 2023 ▸). The apparent scarceness of experimental structures corresponding to *near-predictive* regions may suggest that where a PDB template is used, *AlphaFold* predicts with high confidence (this would be a generally desirable behavior). Alternatively, it may suggest that *near-predictive* residues correlate with regions that are also difficult to solve experimentally (for example conditionally folding).

### 4.5. On ‘hallucinations’

We have intentionally avoided the term ‘hallucination’ in describing low-pLLDT prediction modes. Hallucination implies a prediction gone awry, so if *barbed wire* is indeed a nonpredictive, default behavior, hallucination does not precisely apply. *Pseudostructure* might be understood as a hallucination, having some of the ‘grammar’ of real structure, but little of the meaning. However, *pseudostructure* does not have sole claim to structural hallucinations within *AlphaFold*. Indeed, the most dangerous hallucinations are those that present plausible structures, either in our *near-predictive* mode or in high-pLDDT regions that have the wrong fold despite their confidence. Thus, ‘hallucination’ is a category distinct from but sometimes overlapping with the predictive modes described here.

### 4.6. Testing *AlphaFold*2 prediction behavior

*AlphaFold*’s ability to generate a prediction for any submitted sequence provides an opportunity to construct tests of prediction behavior. In preliminary tests, *AlphaFold*2 predictions of nonsense sequences or text sequences seemed to favor the *pseudostructure* mode, rather than *barbed wire*. This suggests that *barbed wire* prediction is associated with a more specific sequence property than mere randomness. How the density of proline residues in a sequence affects polyproline prediction and prediction behaviors for different kinds of low-complexity sequences could also be tested. However, a thorough exploration of predictions for constructed or random sequences is beyond the scope of this work.

## 5. Conclusion

Development of structure-prediction methods continues to advance. *AlphaFold*3 appears to behave differently from *AlphaFold*2 in low-pLDDT regions (Abramson *et al.*, 2024 ▸). *AlphaFold*3 may assign higher confidence to previous *near-predictive* regions and may be more ambitious in creating folded (possibly hallucinatory) structure in previous *barbed wire* regions. This work was made possible by the availability of proteome-level predictions for *AlphaFold*2. The above descriptions of low-pLDDT behaviors, especially the signature outliers, are therefore specific to *AlphaFold*2 and apply only partially to *AlphaFold*3 or other prediction systems (Baek *et al.*, 2021 ▸; Lin *et al.*, 2023 ▸). However, the Computed Structure Models served by the AlphaFold Protein Structure Database and the RCSB PDB are still *AlphaFold*2 models, and *Phenix* still uses *AlphaFold*2 in its integrated *Predict and Build* pipeline, as does *CCP*4 in its *af-MR* workflow (Simpkin *et al.*, 2023 ▸; Krissinel *et al.*, 2025 ▸). Regardless of the advancements or changes in *AlphaFold*3, *AlphaFold*2 models remain a significant presence in structural biology.

In our work with *MolProbity* structure validation, we have always found visual inspection of models to be a vital part of understanding structures, especially in difficult cases, and *AlphaFold* predictions are by definition difficult cases because their means of production is obscured behind machine learning. The *phenix.barbed_wire_analysis* tool facilitates such an understanding of *AlphaFold*2 predictions.

## Supplementary Material

Supplementary Figures. DOI: 10.1107/S2059798325007843/nz5018sup1.pdf

Numerical results from MobiDB survey. DOI: 10.1107/S2059798325007843/nz5018sup2.xlsx

## Data Availability

The MobiDB survey results are available as an Excel file in the supporting information.
