# Supplementary material for: Categorizing prediction modes within low-pLDDT regions of AlphaFold2 structures: near-predictive, pseudostructure and barbed wire
Source: Acta Crystallogr D Struct Biol. 2025 Sep 12;81(Pt 10):558–72. doi: 10.1107/S2059798325007843 (PMC12485489; doi:10.1107/S2059798325007843)
Supplement: Supplementary file 1 [file d-81-00558-sup1.pdf]

STRUCTURAL
BIOLOGY

Volume 81 (2025)

Supporting information for article:

Categorizing prediction modes within low-pLDDT regions of *AlphaFold*2 structures: near-predictive, pseudostructure and barbed wire

Christopher J. Williams, Vincent B. Chen, David C. Richardson and Jane S. Richardson

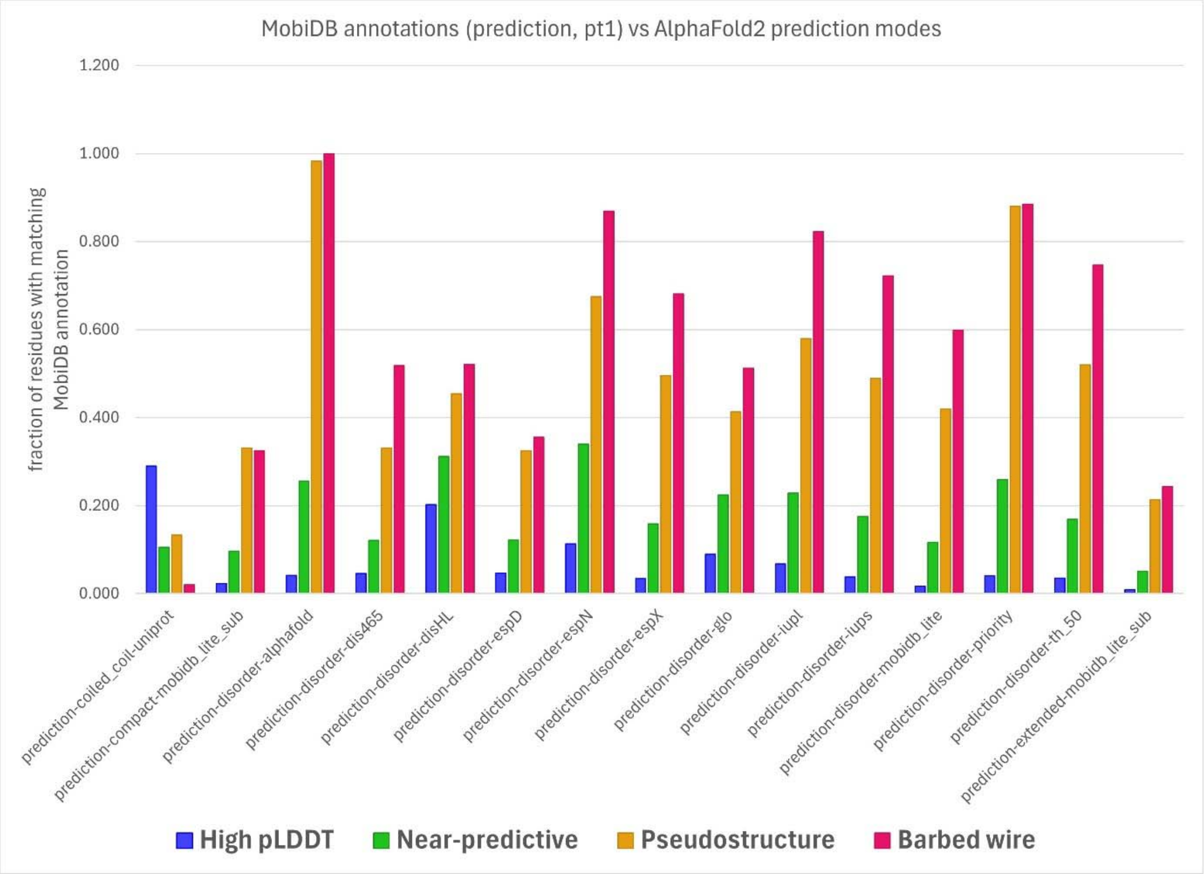

Figure S1 Prediction annotations from MobiDB and their relationships with AlphaFold2 prediction modes. Bar height is the fraction of residues from that prediction mode that were marked with the matching MobiDB annotation. Not all sequences are treated with all annotations, and only residues from duly annotated sequences were considered for each annotation. These prediction annotations show the general stair-step pattern discussed in the main text. Prediction-disorder-iupl is included in Figure 7 as a representative of this behavior. Correlations with prediction-disorder-alphafold are not considered significant, since that annotation is also interpreting AlphaFold results, rather than directly interpreting the underlying sequence.

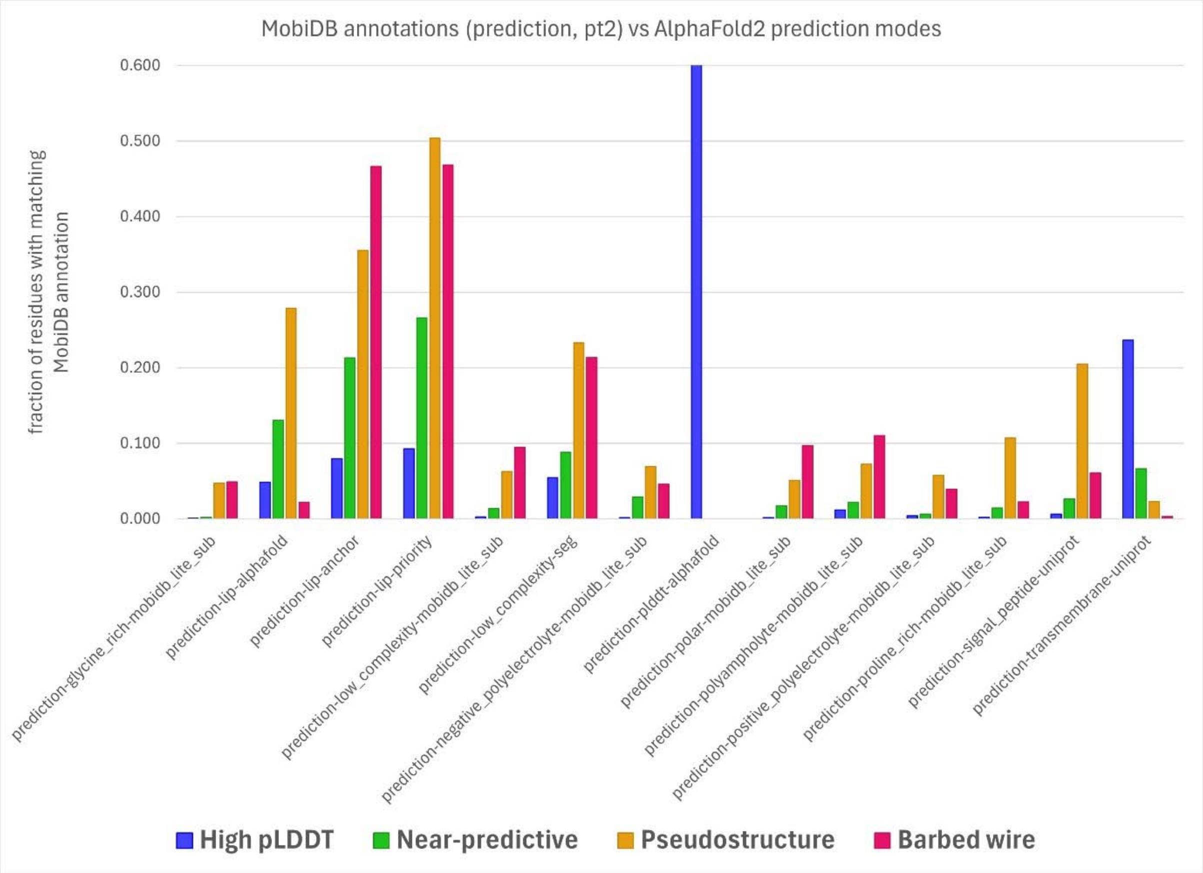

Figure S2 Additional prediction annotations from MobiDB and their relationships with AlphaFold2 prediction modes. Bar height is the fraction of residues from that prediction mode that were marked with the matching MobiDB annotation. Not all sequences are treated with all annotations, and only residues from duly annotated sequences were considered for each annotation. The y-axis is truncated at 0.6, otherwise the trivial prediction-plddt-alphafold result (which goes to 1.0) would dominate. Prediction-low_complexity-seg, prediction-proline_rich-mobidb_lite_sub, and prediction-signal_peptide-uniprot are included in Figure 7. The preference of prediction-transmembrane-uniprot for high-pLDDT residues partly reflects the tendency of membrane insertion helices to be predicted in the *unpacked high-pLDDT* mode, which is included in the high-pLDDT category for these plots.

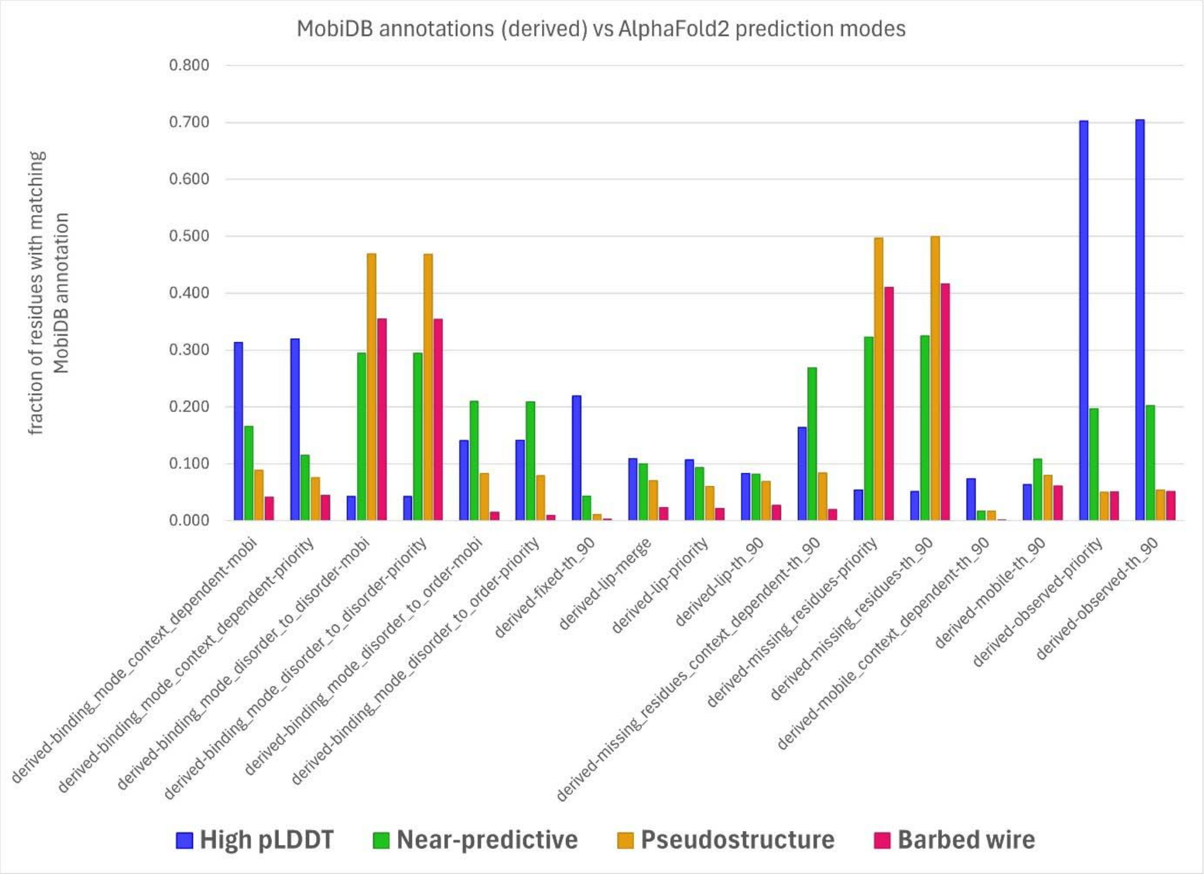

Figure S3 Derived annotations from MobiDB and their relationships with AlphaFold2 prediction modes. Bar height is the fraction of residues from that prediction mode that were marked with the matching MobiDB annotation. Not all sequences are treated with all annotations, and only residues from duly annotated sequences were considered for each annotation. Derived-binding_mode_disorder_to_disorder-mobi and derived-binding_mode_disorder_to_order-mobi are included in Figure 7. Derived-missing_residues-priority is related to residues omitted from experimentally-solved structures. That *near-predictive residues* are frequently missing from their solved structures confirms our difficulty in finding experimentally-solved versions of *near-predictive* regions to check prediction accuracy.

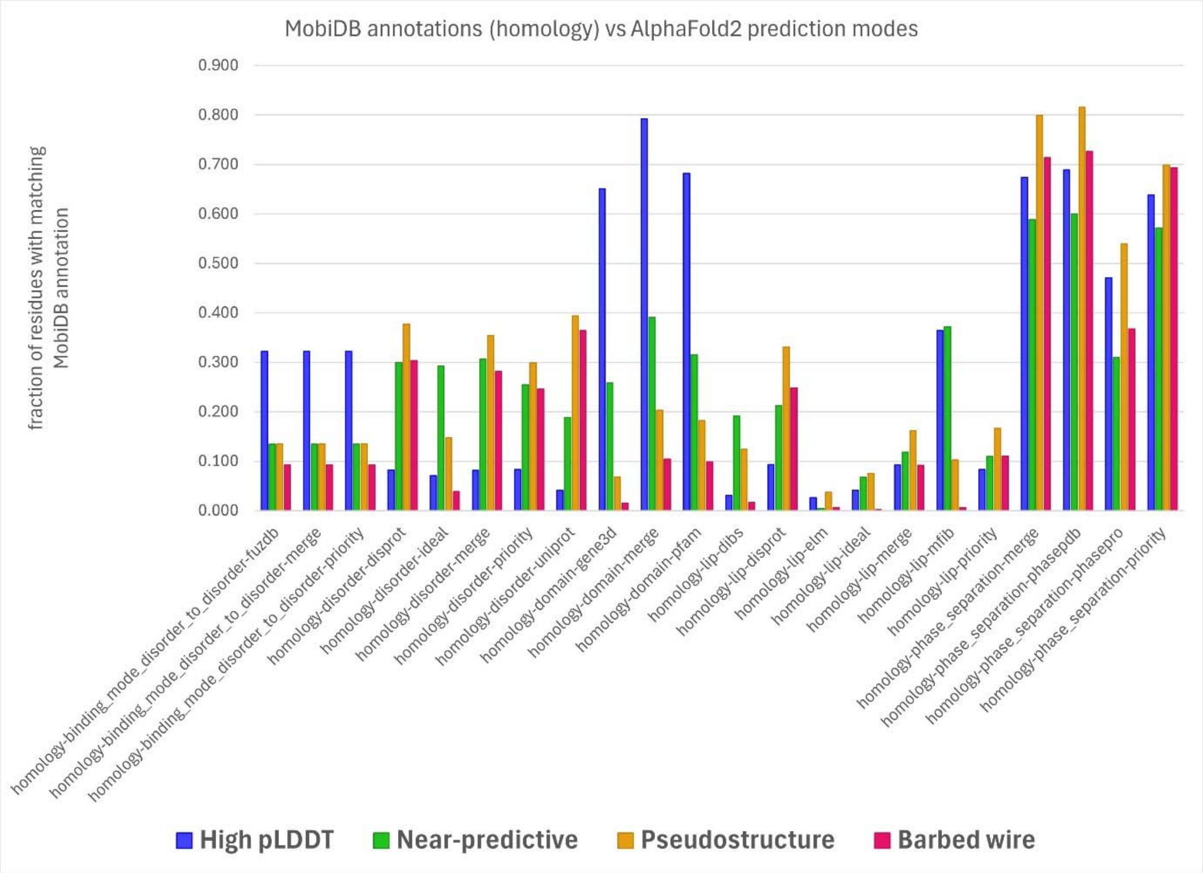

Figure S4 Homology annotations from MobiDB and their relationships with AlphaFold2 prediction modes. Bar height is the fraction of residues from that prediction mode that were marked with the matching MobiDB annotation. Not all sequences are treated with all annotations, and only residues from duly annotated sequences were considered for each annotation. IDEAL is an annotation associated with conditional order; homology-disorder-ideal shows a strong association with *near-predictive*, a lesser association with *pseudostructure*, and very little association with *barbed wire*. This pattern is similar to derived-binding_mode_disorder_to_order-mobi in Figure S3 and supports our conjecture that the *near-predictive* mode is how AlphaFold2 predicts many conditionally ordered IDRs.

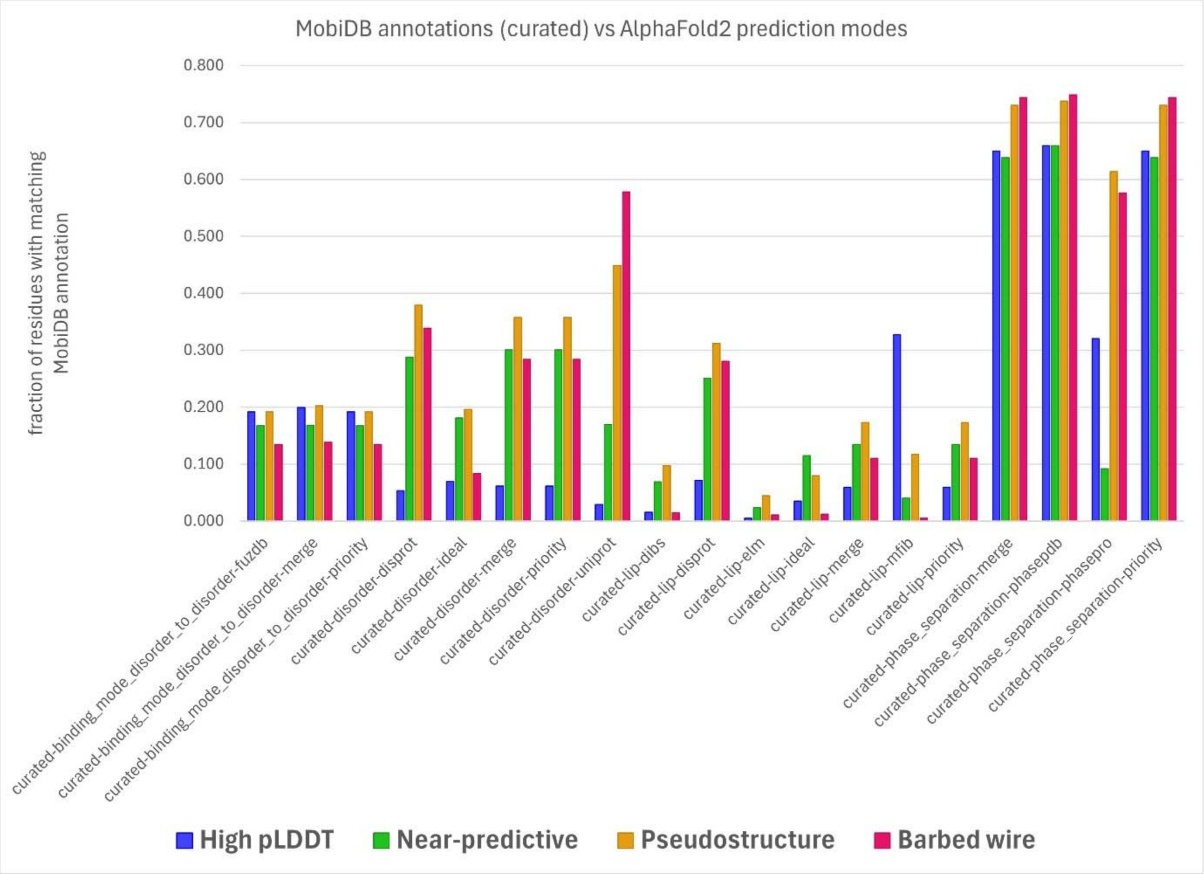

Figure S5 Curated annotations from MobiDB and their relationships with AlphaFold2 prediction modes. Bar height is the fraction of residues from that prediction mode that were marked with the matching MobiDB annotation. Not all sequences are treated with all annotations, and only residues from duly annotated sequences were considered for each annotation. IDEAL is an annotation associated with conditional order. Curated-disorder-ideal shows a more balanced association of *near-predictive* and *pseudostructure* with conditional order than homology-disorder-ideal in Figure S4 above. This may indicate additional complexity in how conditionally folded IDRs manifest in AlphaFold2 predictions, or it may reflect difficulties in IDEAL's literature-based disorder annotation similar to our own challenges in finding experimentally solved *near-predictive* regions.

Note on Figures S6-S20: Not every proteome is well-annotated in MobiDB. Caution – and cross-reference to the supplemental data tables – is advised in interpreting the following plots, especially for *S. aureas* (staa8).

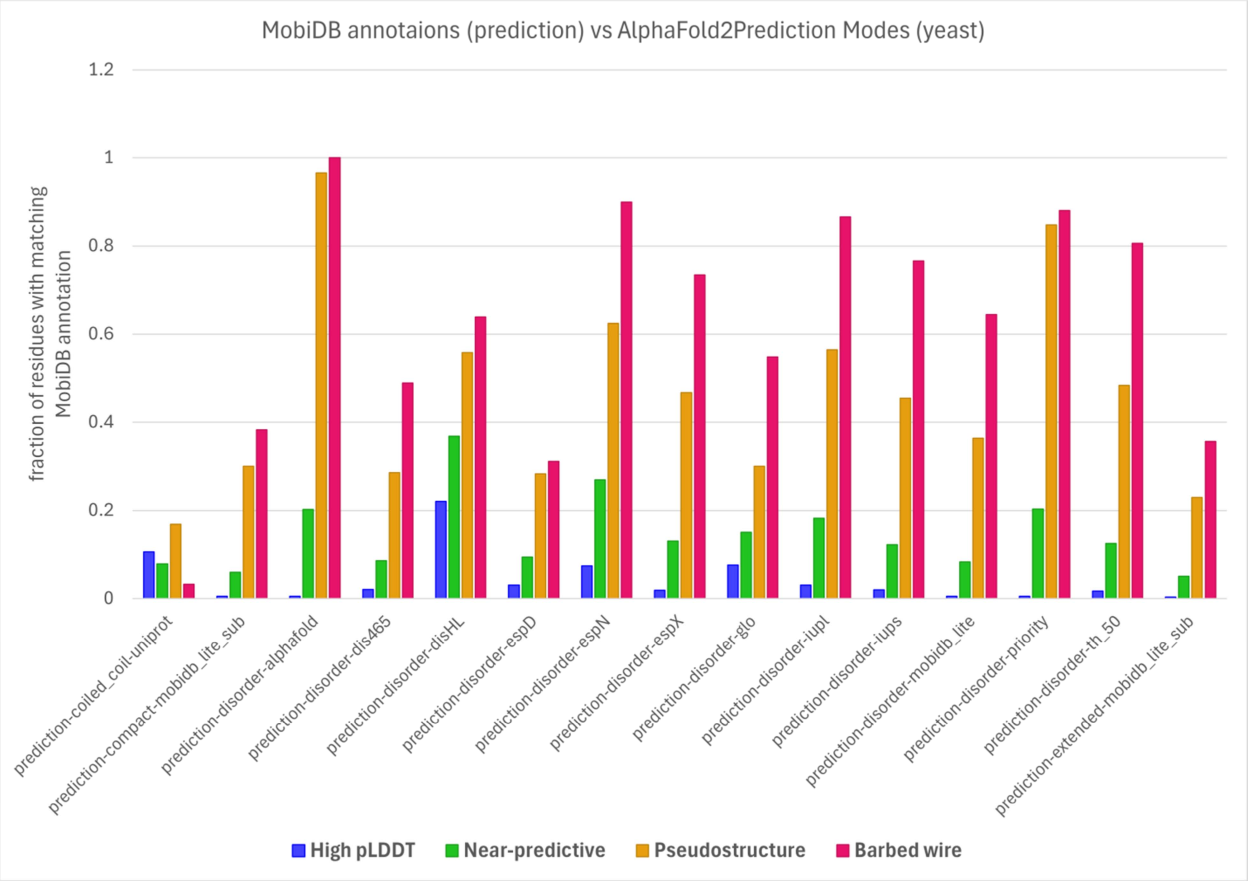

Figure S6 Prediction annotations from MobiDB and their relationships with AlphaFold2 prediction modes in the *S. cerevisiae* proteome. Bar height is the fraction of residues from that prediction mode that were marked with the matching MobiDB annotation. Not all sequences are treated with all annotations, and only residues from duly annotated sequences were considered for each annotation.

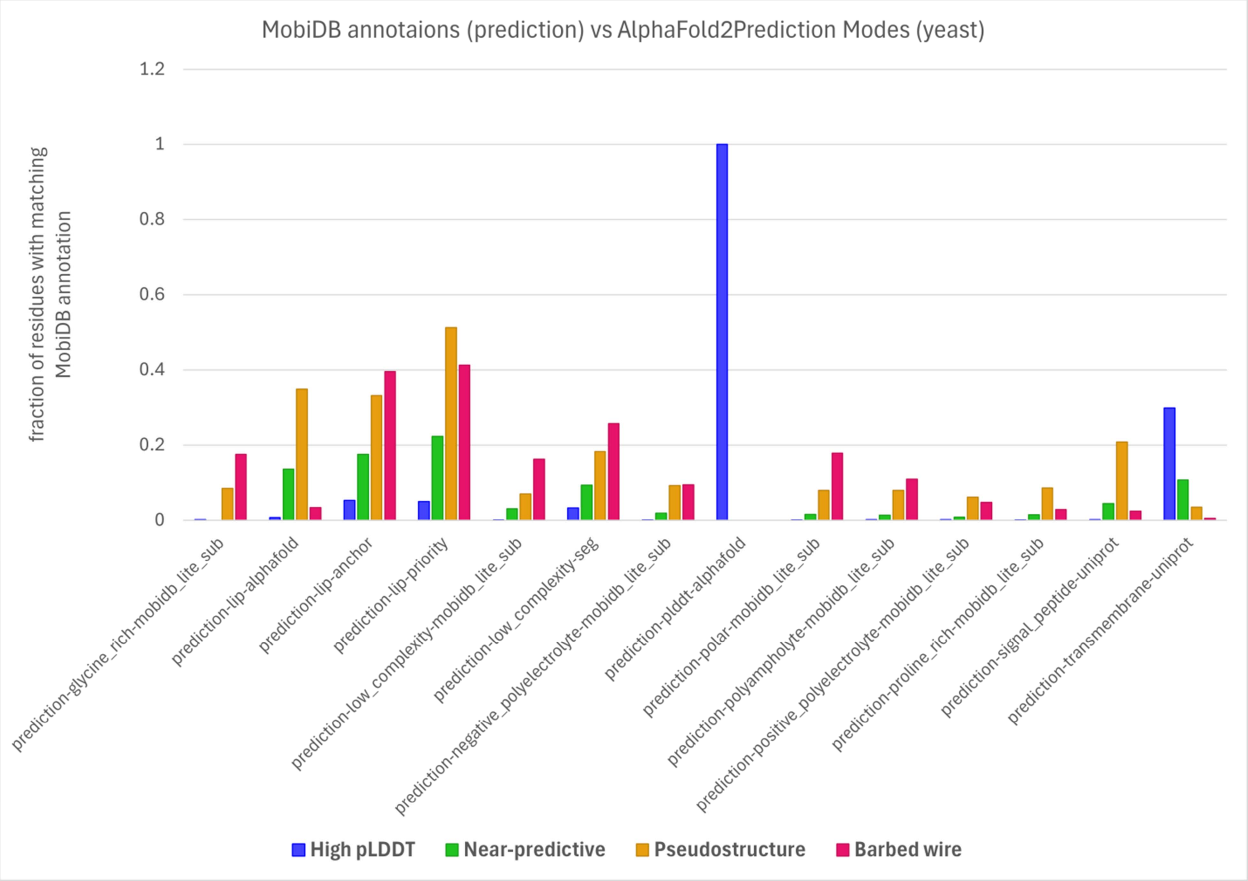

Figure S7 Additional prediction annotations from MobiDB and their relationships with AlphaFold2 prediction modes in the *S. cerevisiae* proteome. Bar height is the fraction of residues from that prediction mode that were marked with the matching MobiDB annotation. Not all sequences are treated with all annotations, and only residues from duly annotated sequences were considered for each annotation.

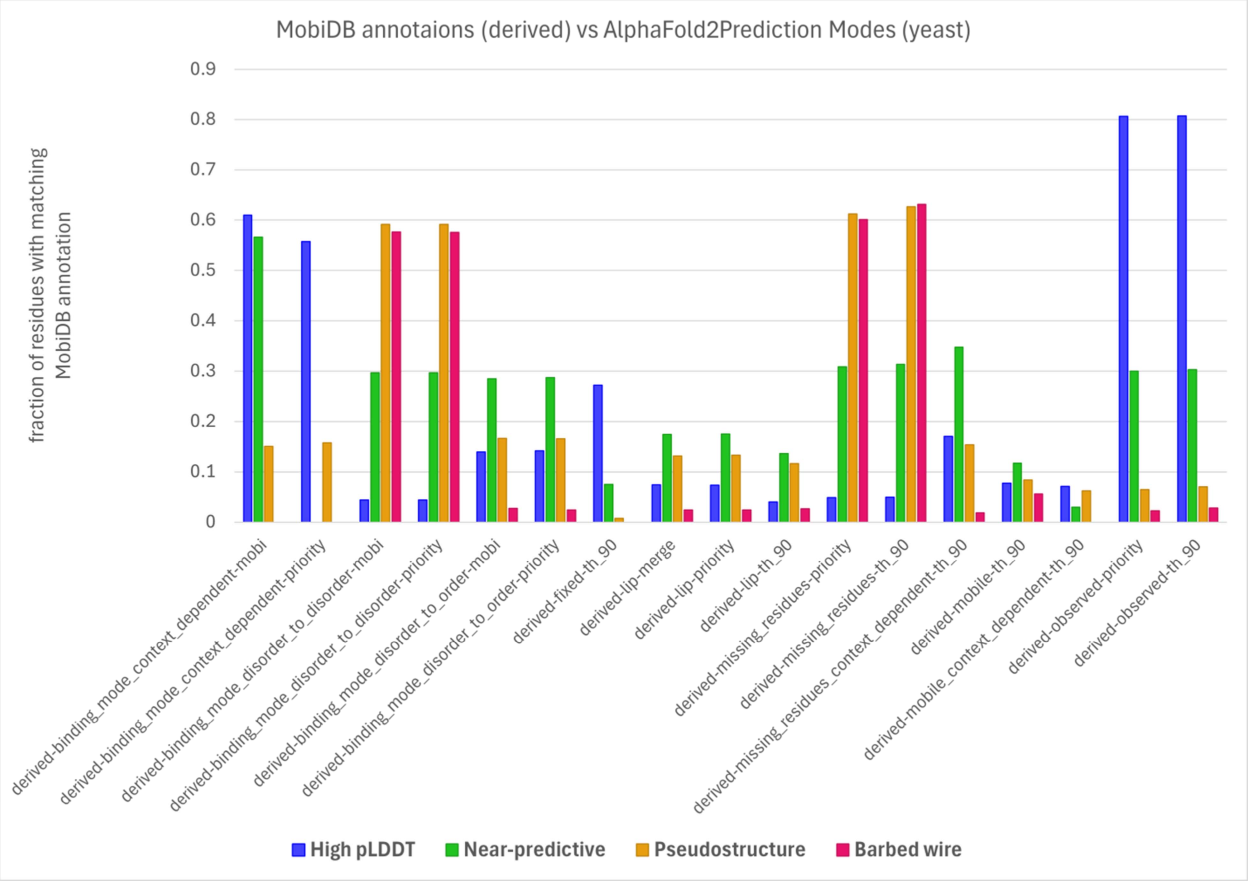

Figure S8 Derived annotations from MobiDB and their relationships with AlphaFold2 prediction modes in the *S. cerevisiae* proteome. Bar height is the fraction of residues from that prediction mode that were marked with the matching MobiDB annotation. Not all sequences are treated with all annotations, and only residues from duly annotated sequences were considered for each annotation.

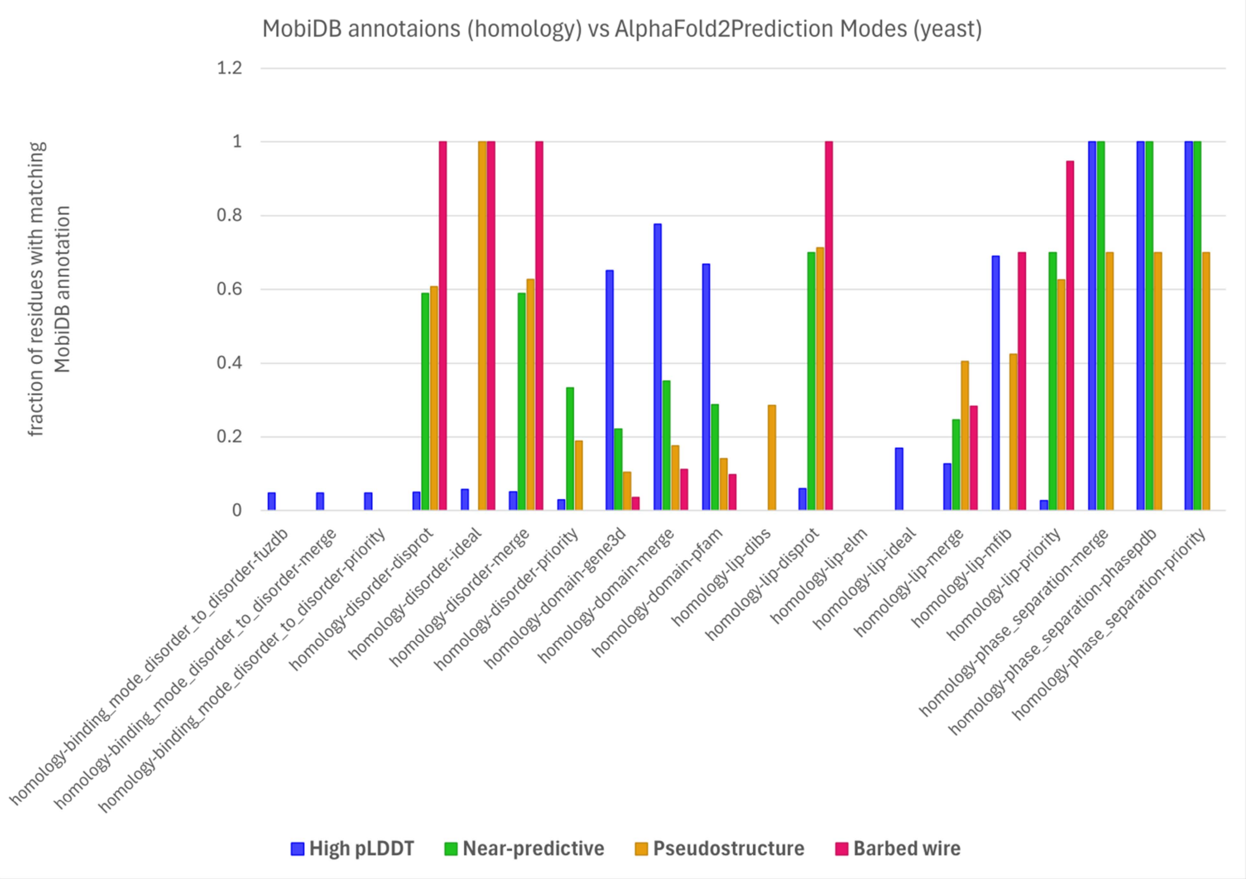

Figure S9 Homology annotations from MobiDB and their relationships with AlphaFold2 prediction modes in the *S. cerevisiae* proteome. Bar height is the fraction of residues from that prediction mode that were marked with the matching MobiDB annotation. Not all sequences are treated with all annotations, and only residues from duly annotated sequences were considered for each annotation.

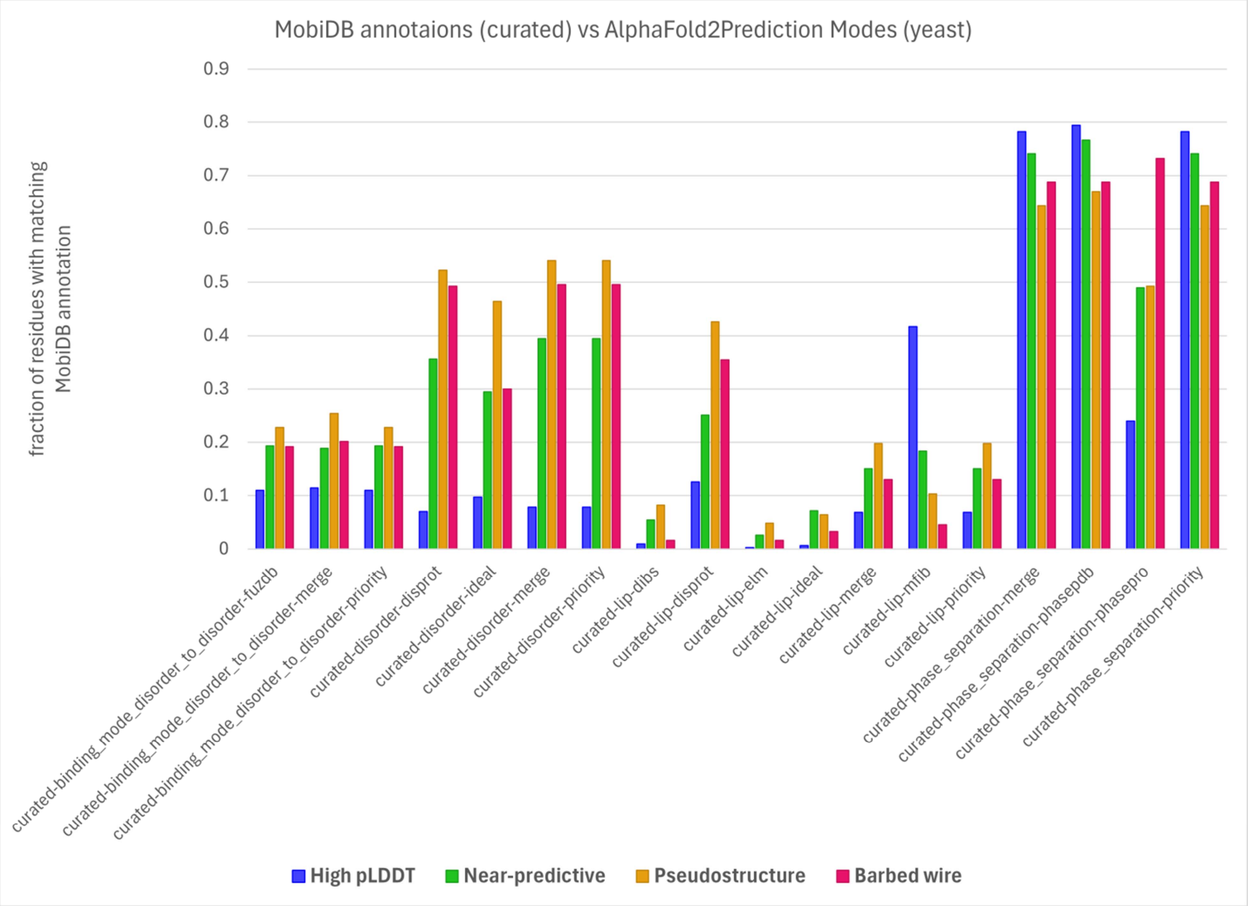

Figure S10 Curated annotations from MobiDB and their relationships with AlphaFold2 prediction modes in the *S. cerevisiae* proteome. Bar height is the fraction of residues from that prediction mode that were marked with the matching MobiDB annotation. Not all sequences are treated with all annotations, and only residues from duly annotated sequences were considered for each annotation.

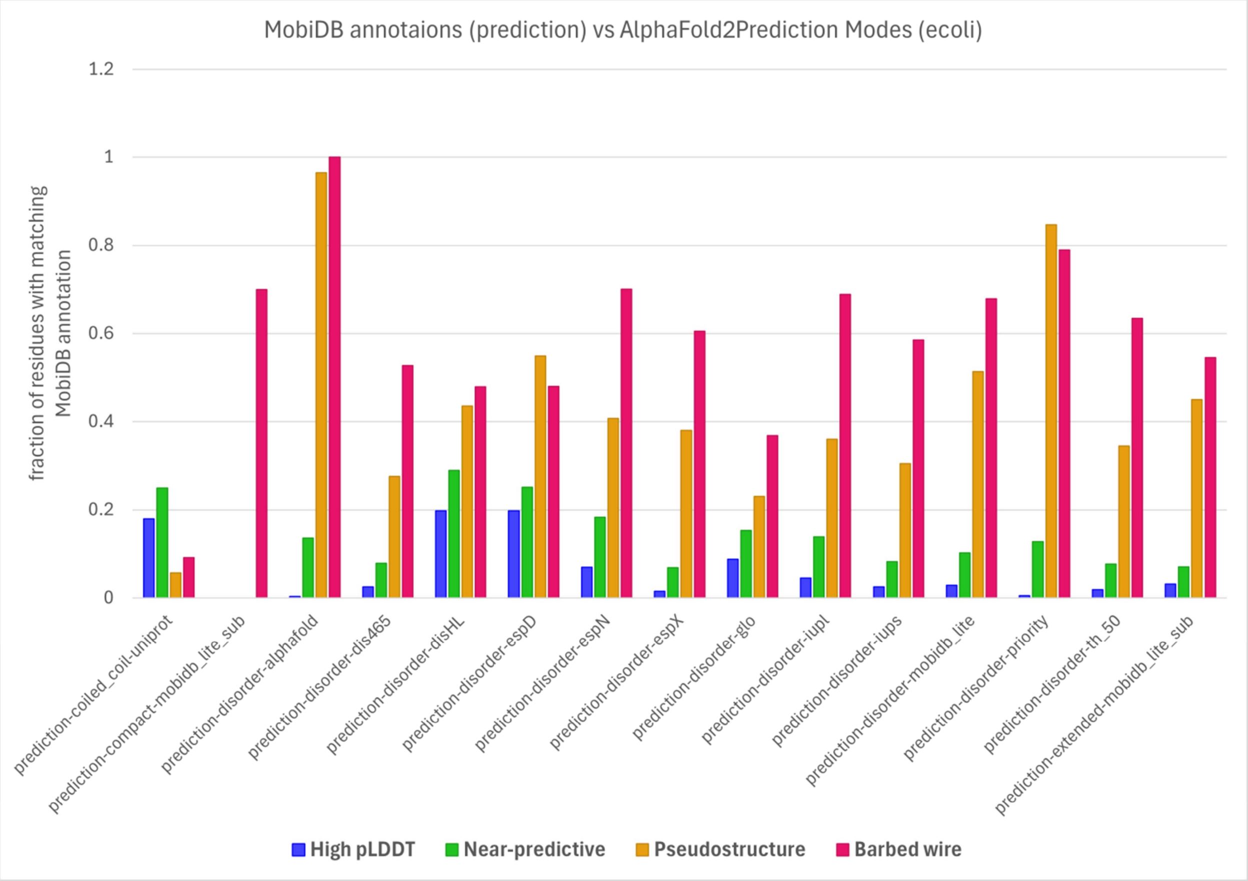

Figure S11 Prediction annotations from MobiDB and their relationships with AlphaFold2 prediction modes in the *E. coli* proteome. Bar height is the fraction of residues from that prediction mode that were marked with the matching MobiDB annotation. Not all sequences are treated with all annotations, and only residues from duly annotated sequences were considered for each annotation.

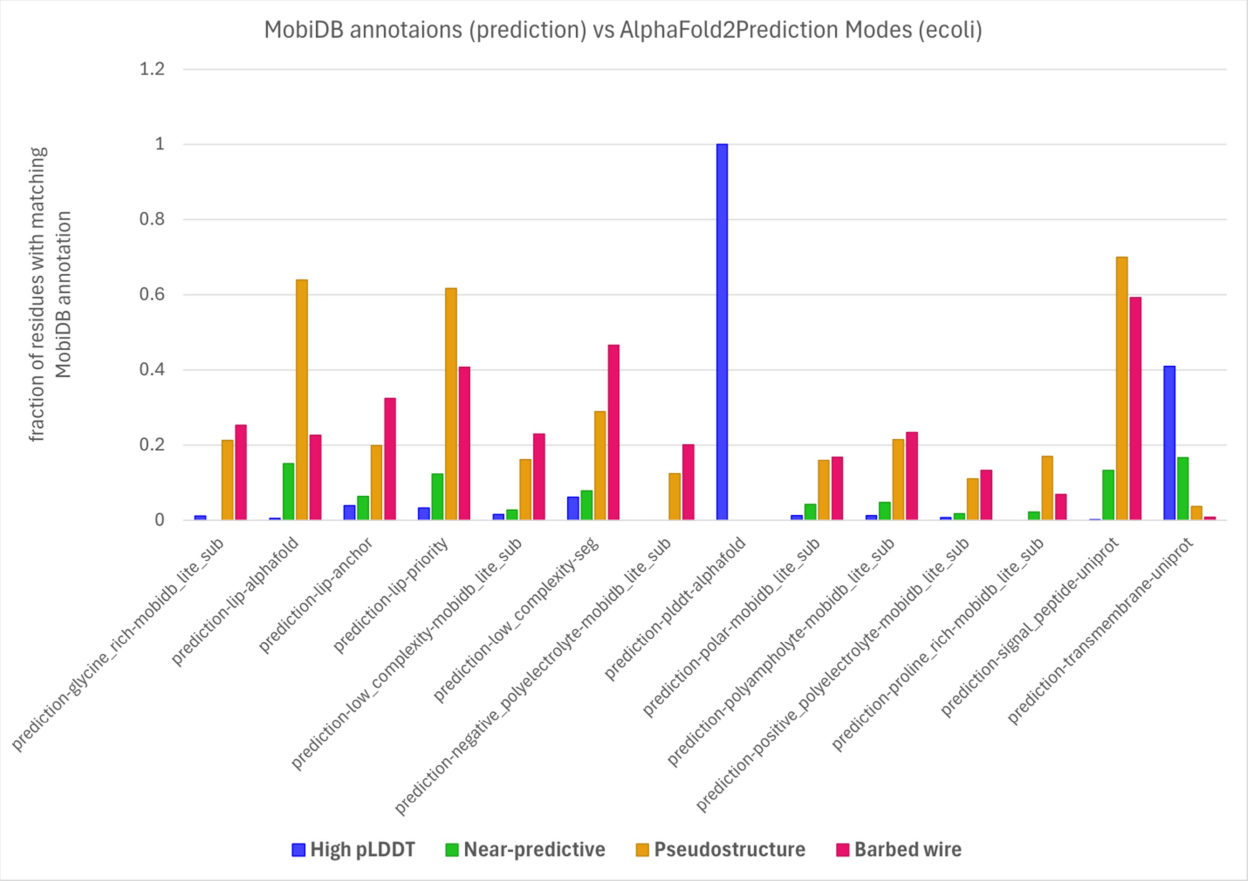

Figure S12 Additional prediction annotations from MobiDB and their relationships with AlphaFold2 prediction modes in the *E. coli* proteome. Bar height is the fraction of residues from that prediction mode that were marked with the matching MobiDB annotation. Not all sequences are treated with all annotations, and only residues from duly annotated sequences were considered for each annotation.

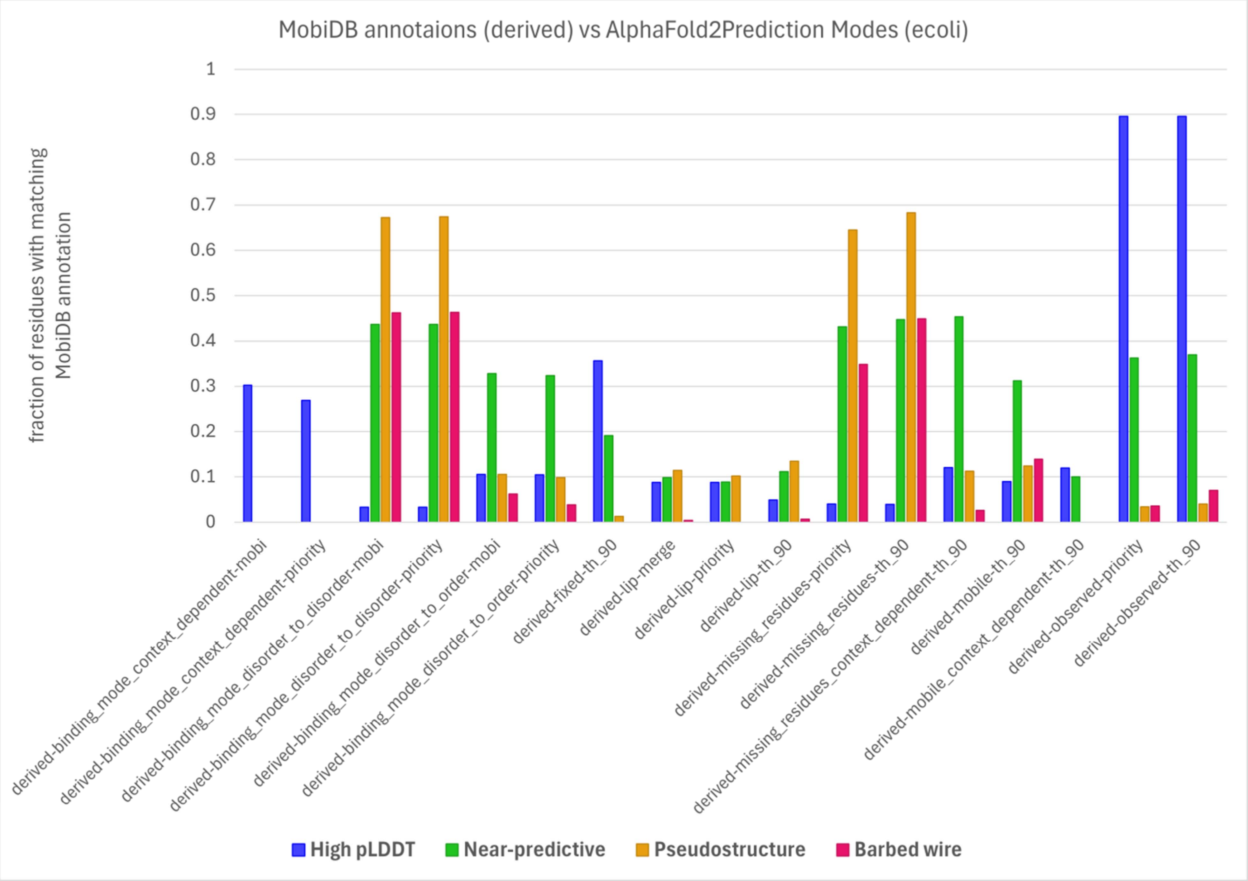

Figure S13 Derived annotations from MobiDB and their relationships with AlphaFold2 prediction modes in the *E. coli* proteome. Bar height is the fraction of residues from that prediction mode that were marked with the matching MobiDB annotation. Not all sequences are treated with all annotations, and only residues from duly annotated sequences were considered for each annotation.

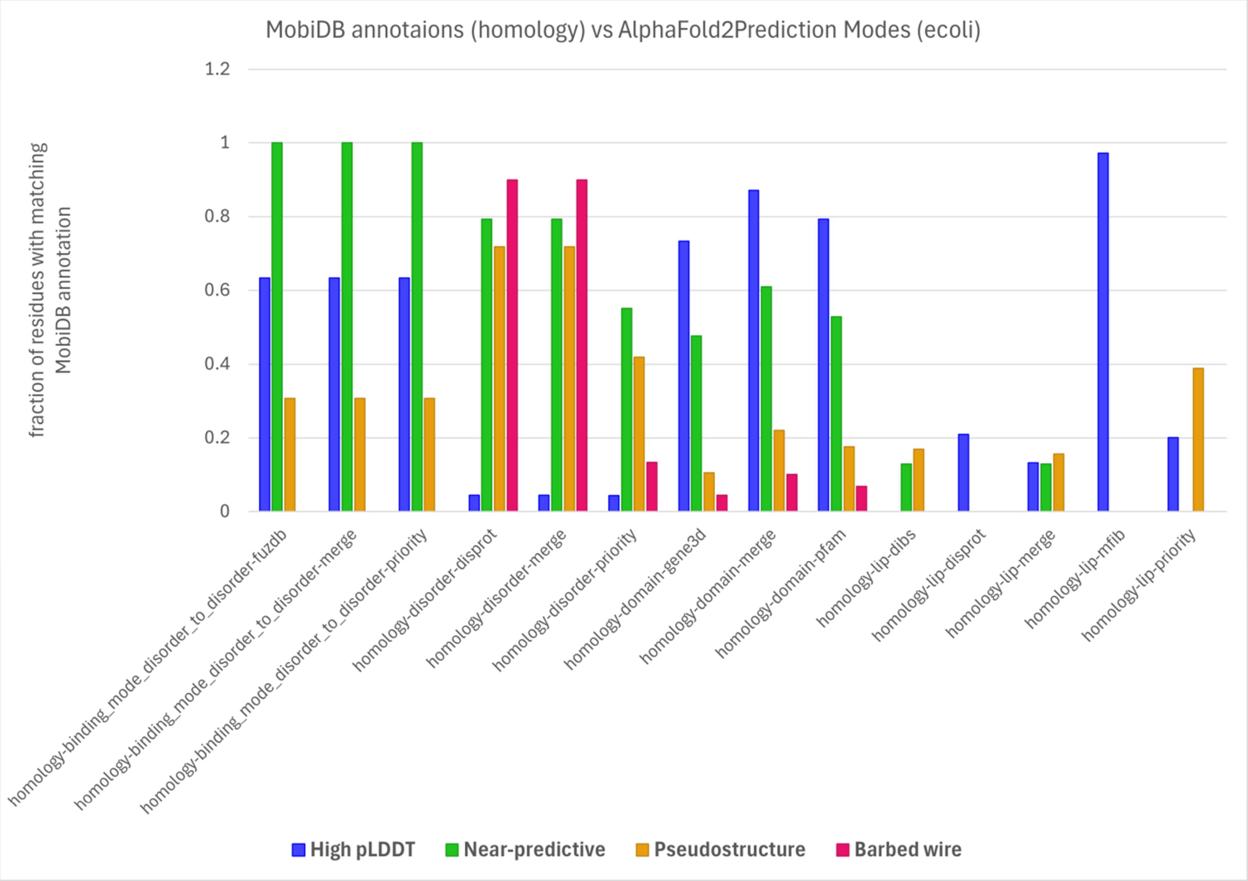

Figure S14 Homology annotations from MobiDB and their relationships with AlphaFold2 prediction modes in the *E. coli* proteome. Bar height is the fraction of residues from that prediction mode that were marked with the matching MobiDB annotation. Not all sequences are treated with all annotations, and only residues from duly annotated sequences were considered for each annotation.

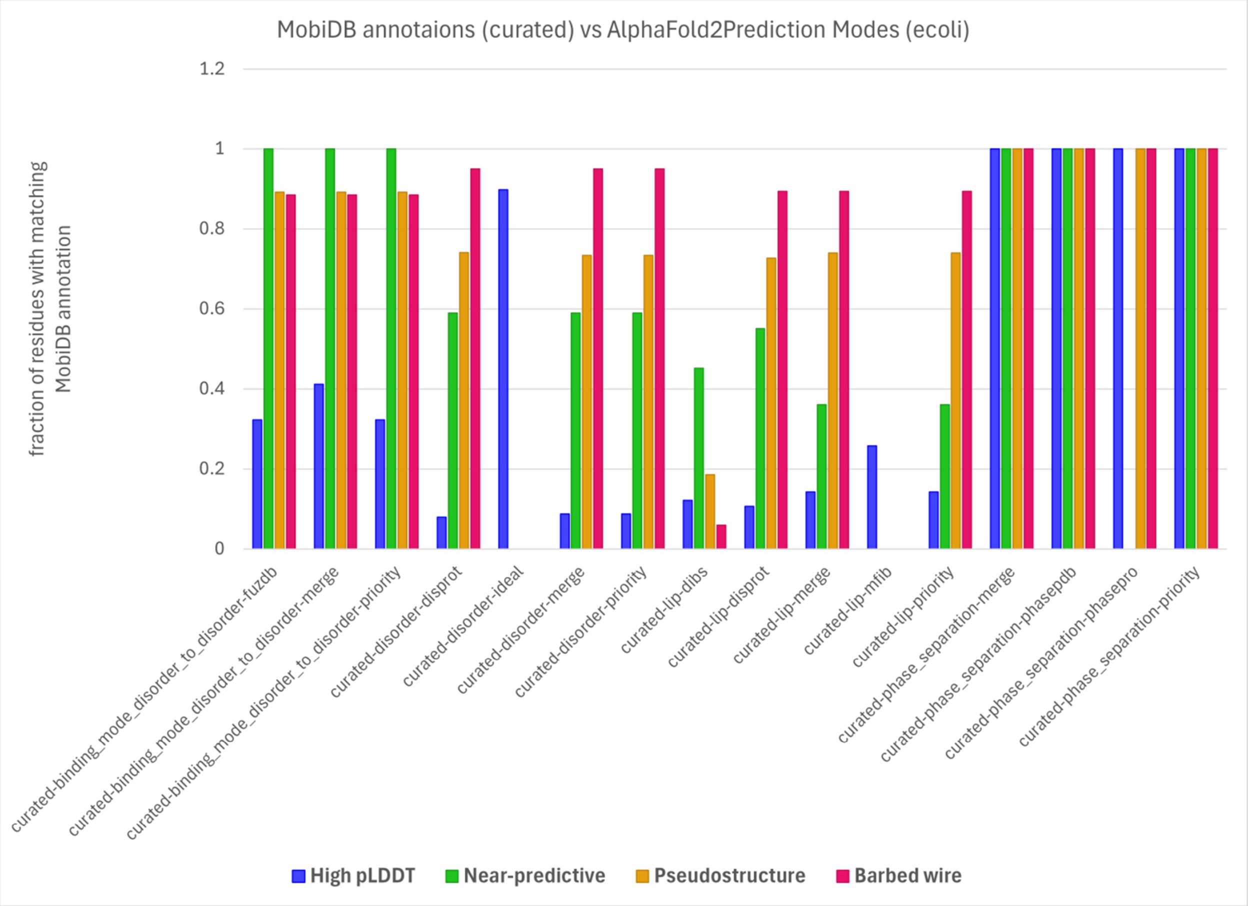

Figure S15 Curated annotations from MobiDB and their relationships with AlphaFold2 prediction modes in the *E. coli* proteome. Bar height is the fraction of residues from that prediction mode that were marked with the matching MobiDB annotation. Not all sequences are treated with all annotations, and only residues from duly annotated sequences were considered for each annotation.

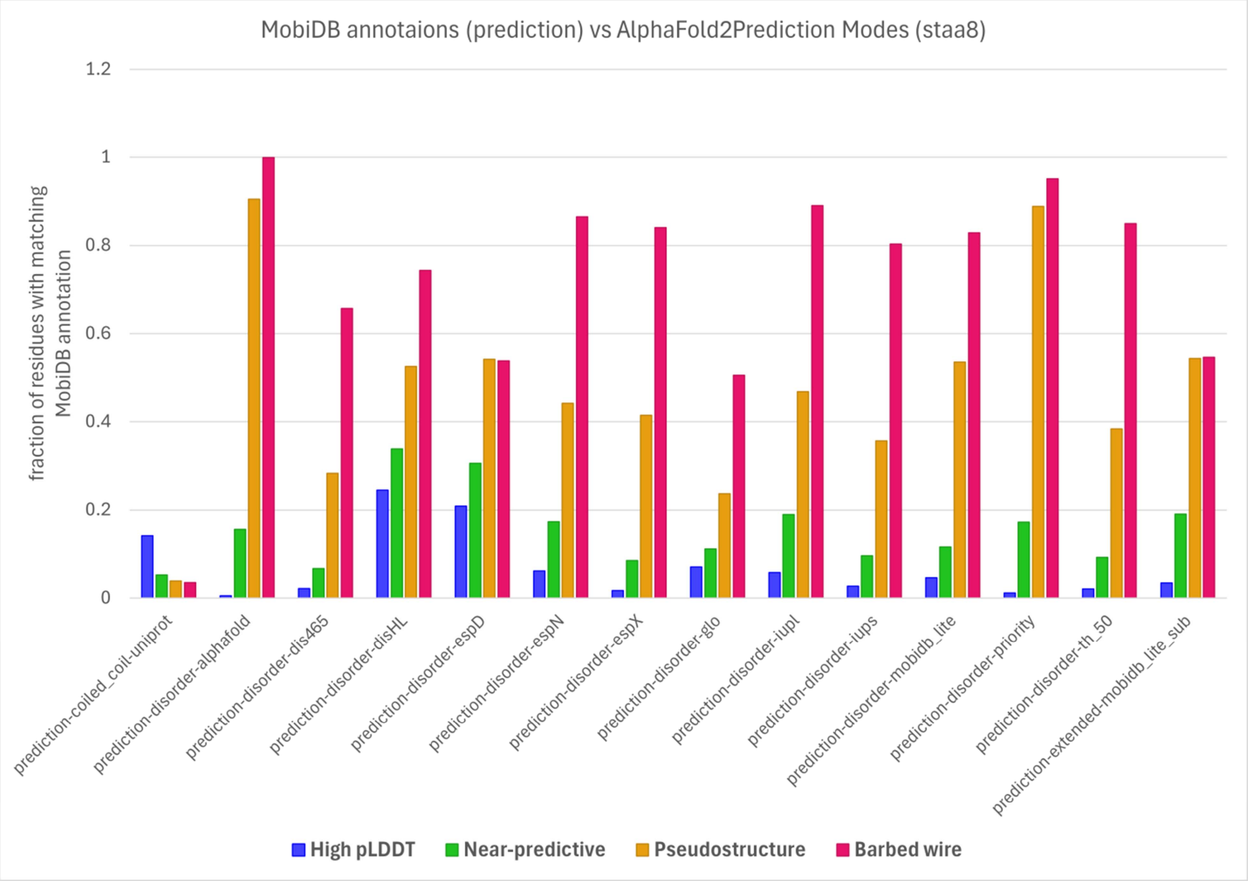

Figure S16 Prediction annotations from MobiDB and their relationships with AlphaFold2 prediction modes in the *S. aureas* proteome. Bar height is the fraction of residues from that prediction mode that were marked with the matching MobiDB annotation. Not all sequences are treated with all annotations, and only residues from duly annotated sequences were considered for each annotation.

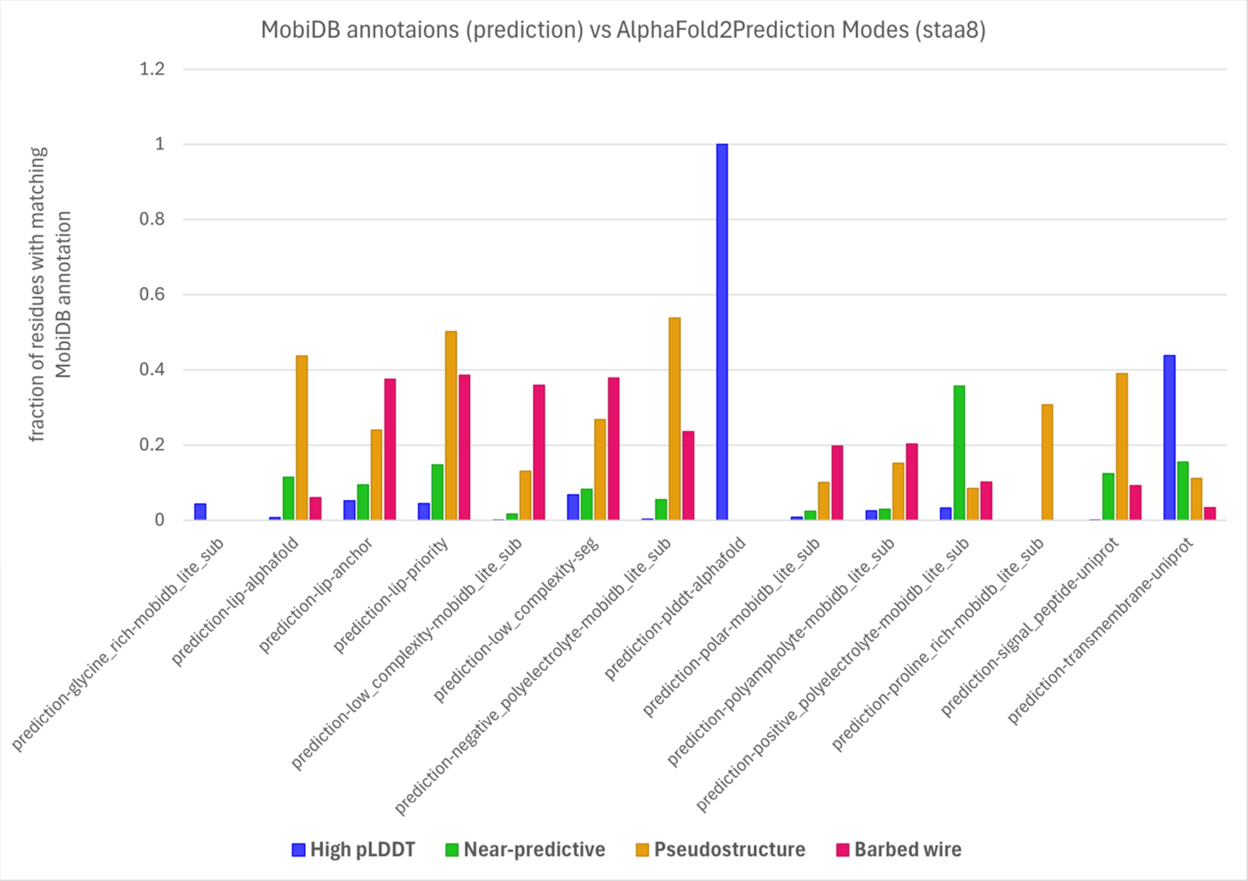

Figure S17 Additional prediction annotations from MobiDB and their relationships with AlphaFold2 prediction modes in the *S. aureas* proteome. Bar height is the fraction of residues from that prediction mode that were marked with the matching MobiDB annotation. Not all sequences are treated with all annotations, and only residues from duly annotated sequences were considered for each annotation.

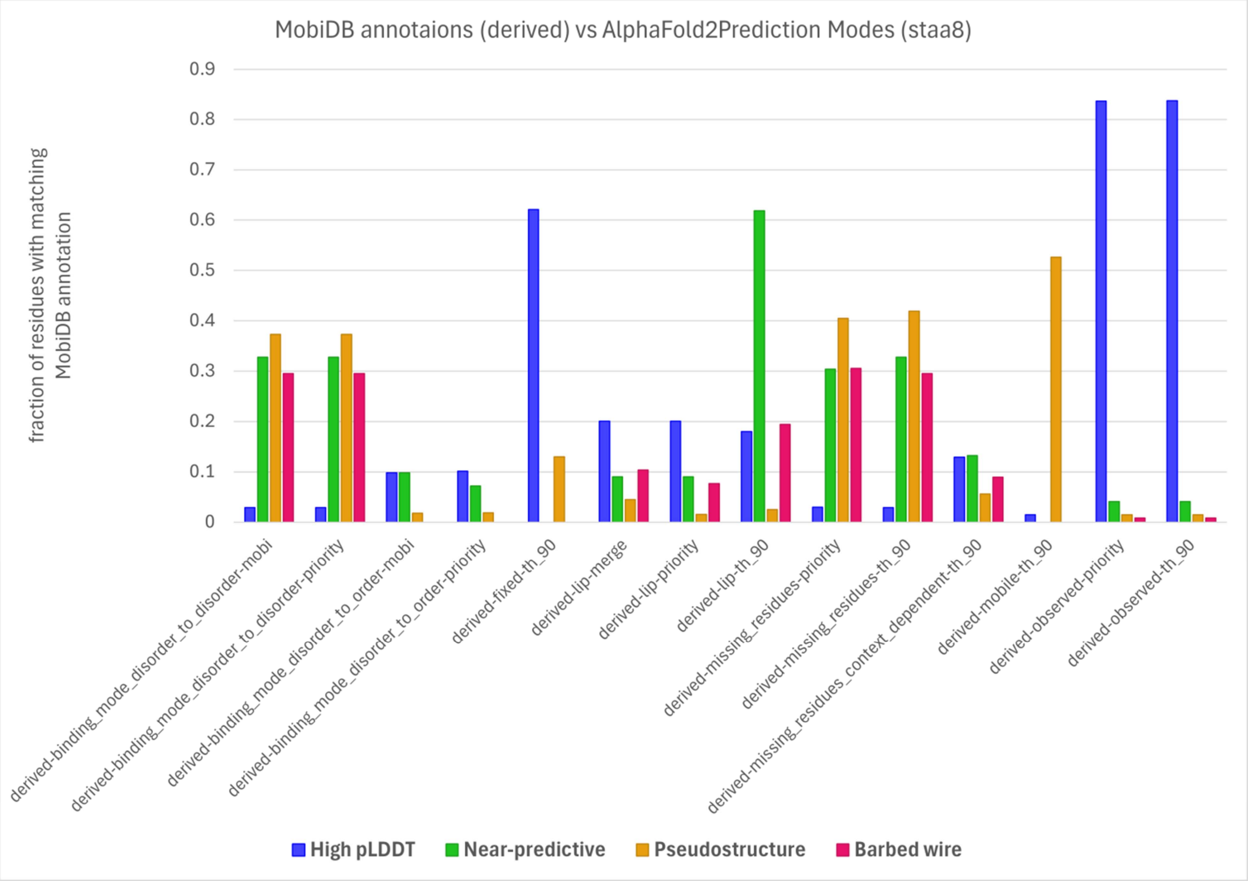

Figure S18 Derived annotations from MobiDB and their relationships with AlphaFold2 prediction modes in the *S. aureas* proteome. Bar height is the fraction of residues from that prediction mode that were marked with the matching MobiDB annotation. Not all sequences are treated with all annotations, and only residues from duly annotated sequences were considered for each annotation.

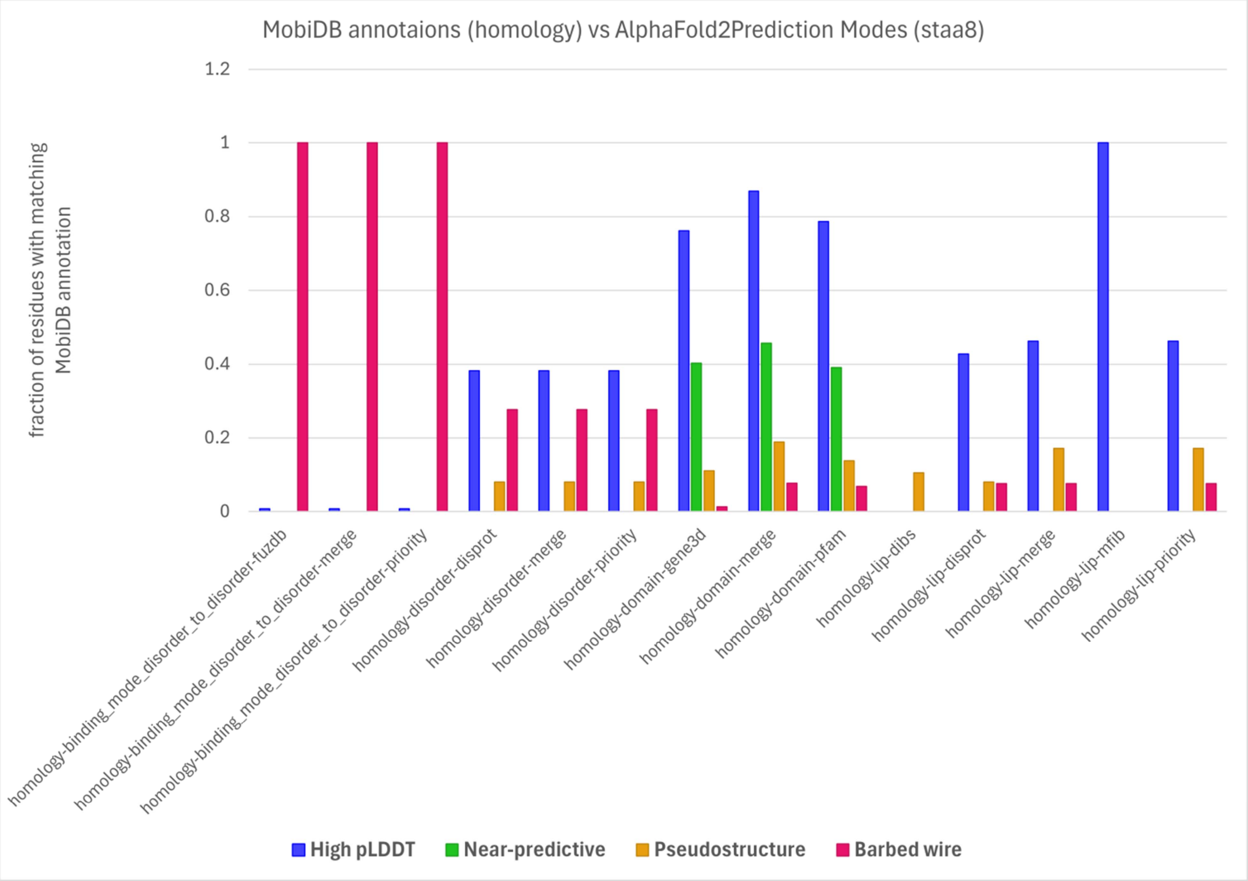

Figure S19 Homology annotations from MobiDB and their relationships with AlphaFold2 prediction modes in the *S. aureas* proteome. Bar height is the fraction of residues from that prediction mode that were marked with the matching MobiDB annotation. Not all sequences are treated with all annotations, and only residues from duly annotated sequences were considered for each annotation.

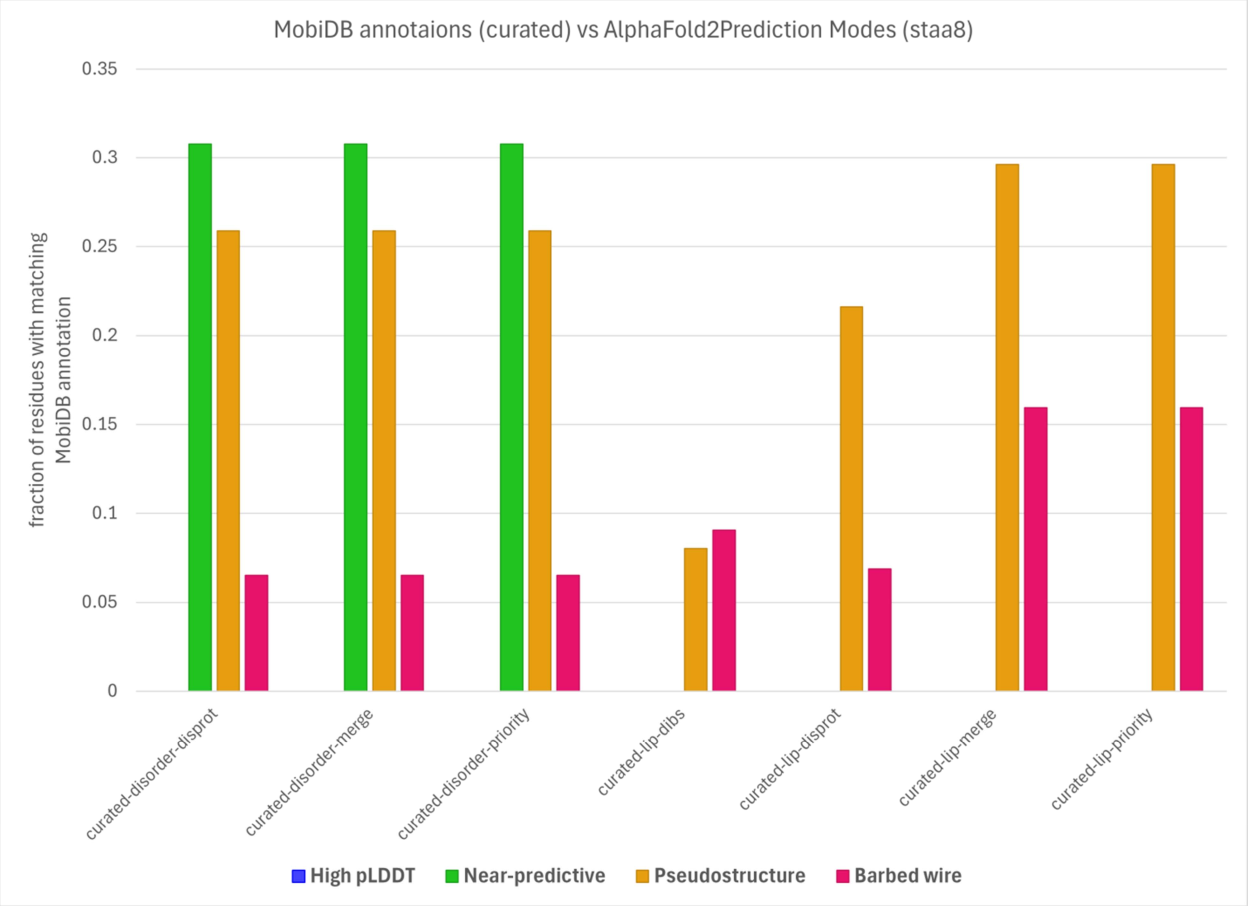

Figure S20 Curated annotations from MobiDB and their relationships with AlphaFold2 prediction modes in the *S. aureas* proteome. Bar height is the fraction of residues from that prediction mode that were marked with the matching MobiDB annotation. Not all sequences are treated with all annotations, and only residues from duly annotated sequences were considered for each annotation.

Interpretation of curated disorder metrics for *S. aureas* is severely impeded by limited annotation in MobiDB. This plot is included only for completeness.

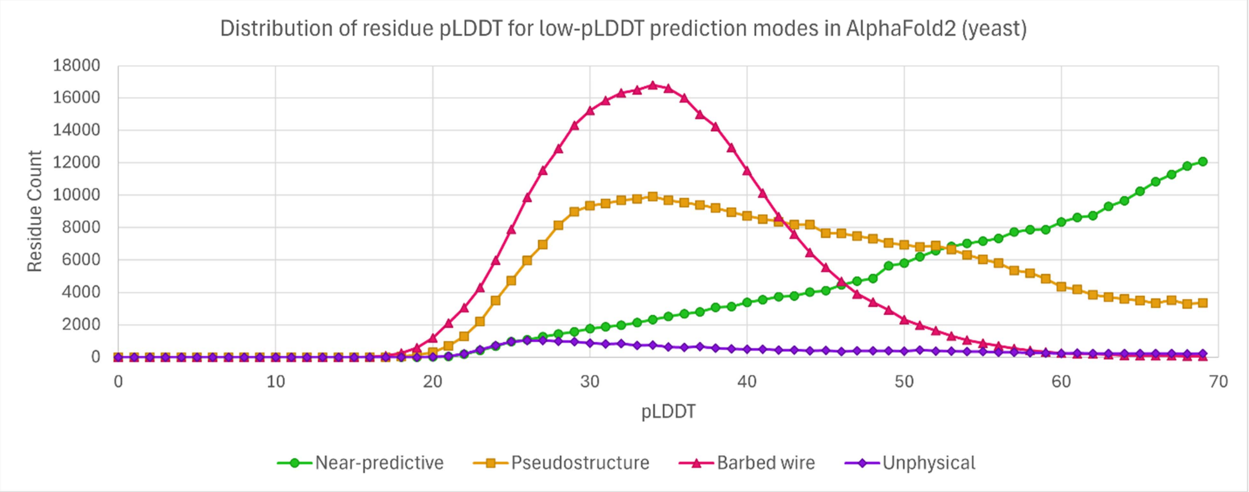

Figure S21 pLDDT distributions for low-pLDDT prediction modes, pLDDT bins of 1, for sequences from the *Saccharomyces cerevisiae* proteome.

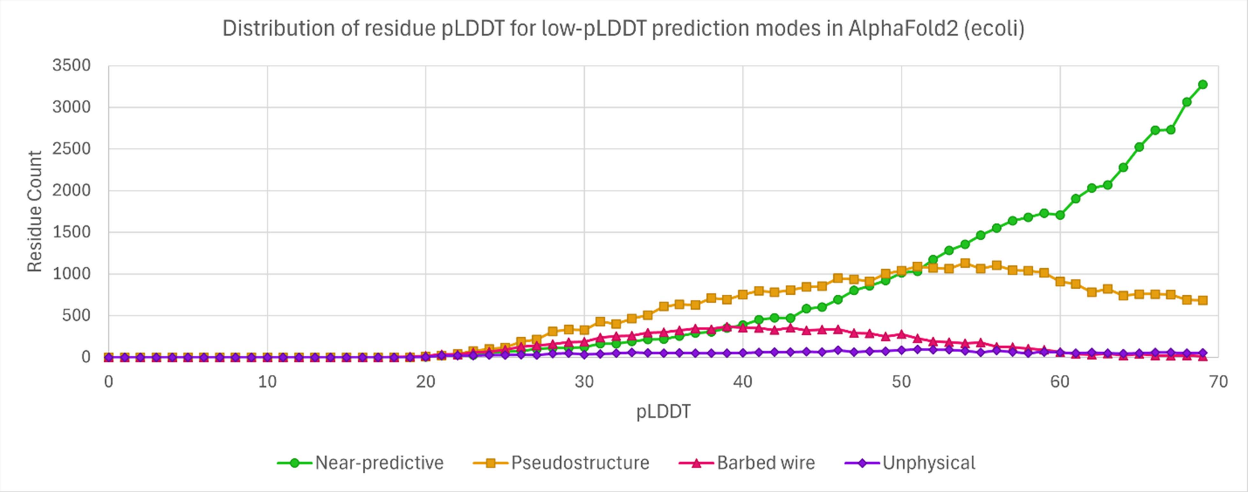

Figure S22 pLDDT distributions for low-pLDDT prediction modes, pLDDT bins of 1, for sequences from the *Escherichia coli* proteome.

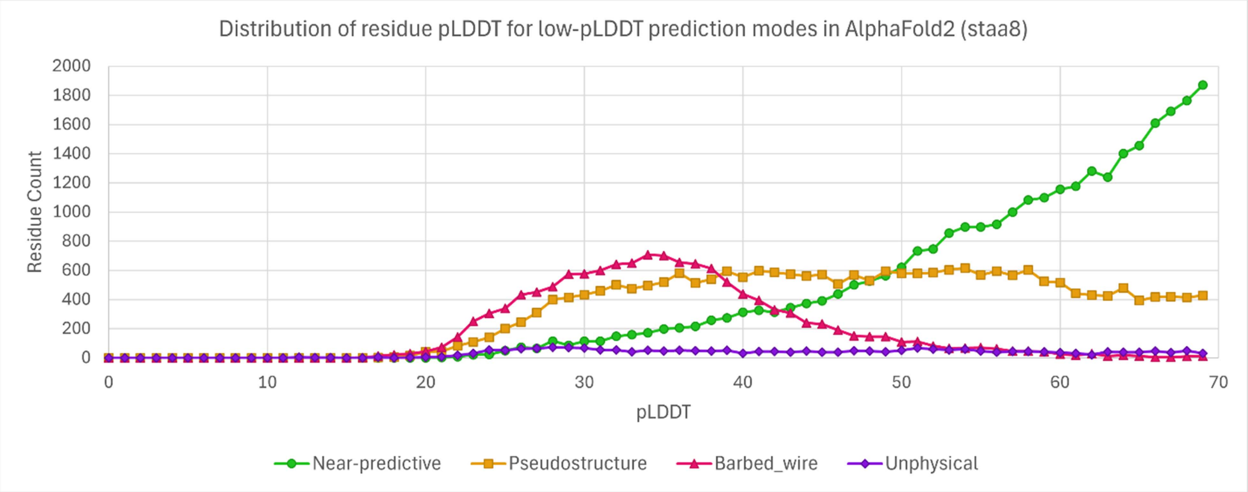

Figure S23 pLDDT distributions for the major low-pLDDT prediction modes, pLDDT bins of 1, for sequences from the *Staphylococcus aureas* proteome.

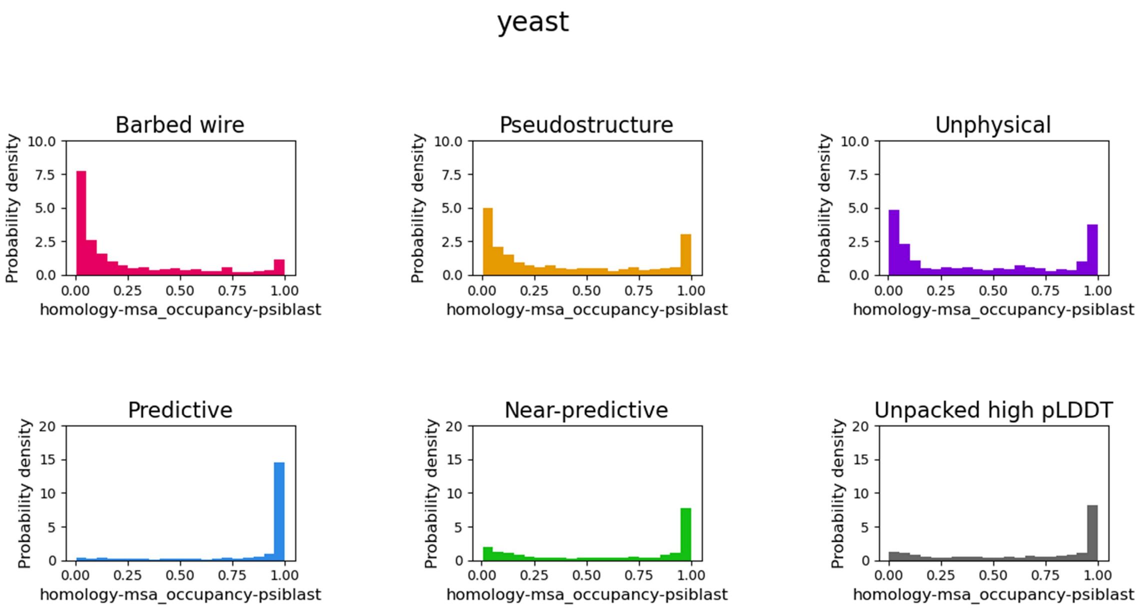

Figure S24 Multiple sequence alignment occupancy histogram distributions for AlphaFold2 *S. cerevisiae* proteome predictions, as annotated in MobiDB. Groupings are similar to those seen in the human proteome. The three non-predictive modes (*barbed wire*, *pseudostructure*, and *unphysical*) show similar distributions to each other. *Near-predictive* has a distribution more similar to the high-pLDDT modes, supporting our association of *near-predictive* regions with *predictive*. Similarity between *near-predictive* and *unpacked high-pLDDT* suggests that *near-predictive* also contains conditionally binding regions.

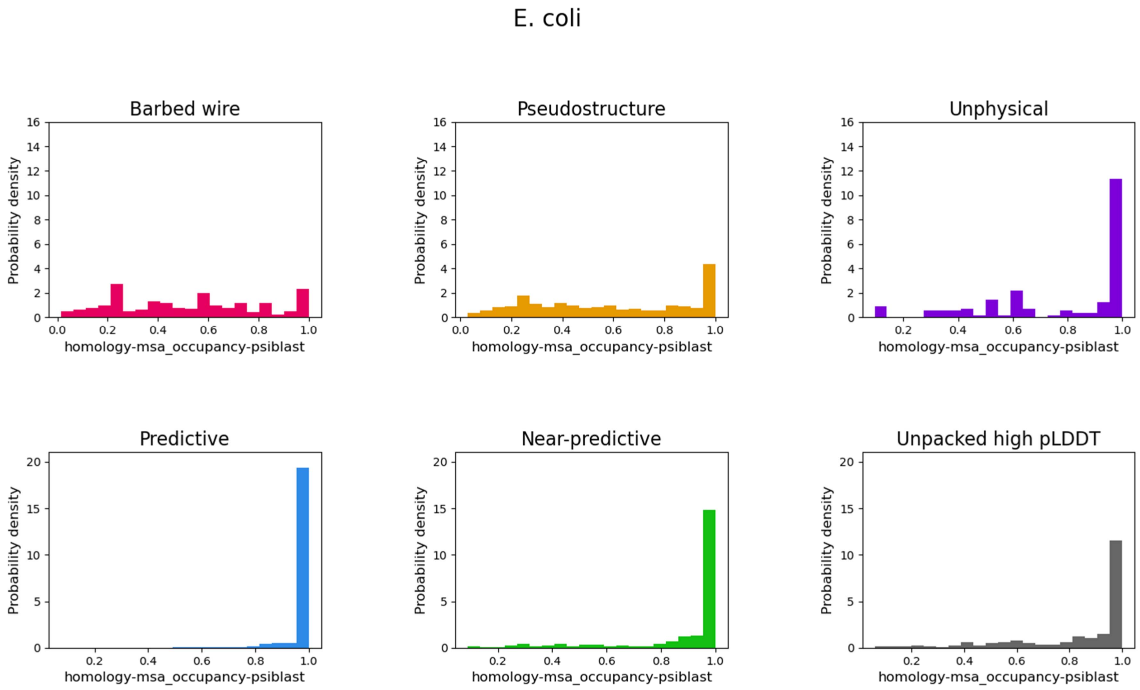

Figure S25 Multiple sequence alignment occupancy histogram distributions for AlphaFold2 *E. coli* proteome predictions, as annotated in MobiDB. Groupings are similar to those seen in the human proteome. The three non-predictive modes (*barbed wire*, *pseudostructure*, and *unphysical*) show similar distributions to each other. *Near-predictive* has a distribution more similar to the high-pLDDT modes, supporting our association of *near-predictive* regions with *predictive*. Similarity between *near-predictive* and *unpacked high-pLDDT* suggests that *near-predictive* also contains conditionally binding regions. Homology-msa-occupancy-psiblast annotations for *S. aureas* were too sparse (0.3% of sequences) to produce meaningful plots.
